# Electric recycling of Portland cement at scale

**DOI:** 10.1038/s41586-024-07338-8

**Published:** 2024-05-22

**Authors:** Cyrille F. Dunant, Shiju Joseph, Rohit Prajapati, Julian M. Allwood

**Affiliations:** Department of Engineering, University of Cambridge, Cambridge, UK

**Keywords:** Civil engineering, Ceramics

## Abstract

Cement production causes 7.5% of global anthropogenic CO_2_ emissions, arising from limestone decarbonation and fossil-fuel combustion^1–3^. Current decarbonation strategies include substituting Portland clinker with supplementary materials, but these mainly arise in emitting processes, developing alternative binders but none yet promises scale, or adopting carbon capture and storage that still releases some emissions^4–8^. However, used cement is potentially an abundant, decarbonated feedstock. Here we show that recovered cement paste can be reclinkered if used as a partial substitute for the lime–dolomite flux used in steel recycling nowadays. The resulting slag can meet existing specifications for Portland clinker and can be blended effectively with calcined clay and limestone. The process is sensitive to the silica content of the recovered cement paste, and silica and alumina that may come from the scrap, but this can be adjusted easily. We show that the proposed process may be economically competitive, and if powered by emissions-free electricity, can lead to zero emissions cement while also reducing the emissions of steel recycling by reducing lime flux requirements. The global supply of scrap steel for recycling may treble by 2050, and it is likely that more slag can be made per unit of steel recycled. With material efficiency in construction^9,10^, future global cement requirements could be met by this route.

## Abstract (web summary)

Recovered cement paste can be reclinkered if used as a partial substitute for the lime–dolomite flux used in steel recycling, which can reduce waste and carbon emissions.

## Main

Concrete is the most used material on the planet, after water, and is responsible for approximately 7.5% of total anthropogenic CO_2_ emissions^1–3^.

The two main strategies deployed to date to reduce emissions from Portland cement production are fuel switching (using fossil gas, municipal waste or biofuels instead of coke and petcoke) and increased use of substitution materials, such as fly ash and slag. However, these strategies cannot lead to zero emissions. Moreover, the two most common substitution materials, ground-granulated blast furnace slag and fly ash are themselves by-products of primary steel production and coal power stations, both of which are highly emitting and therefore must be phased out in the transition to zero emissions. There has been a recent surge of interest in ternary blends with calcined clay and limestone (commonly known as LC^3^)^4^. Nevertheless, it could be difficult to achieve substitution levels greater than 50% as these blends still require Portland clinker for activation^5^. Meanwhile, alternative clinkers or binder systems to replace Portland clinker are under development, but none so far can be made at scale without significant emissions^6–8^. Carbon capture and storage could potentially be applied to cement making^11,12^ but current projects attempt the capture of the process emissions only, without storage^13^.

Most emissions associated with concrete arise in the production of Portland cement, about 40% from fuel combustion and about 60% from limestone decarbonation. Both sources are hard to abate. High-temperature processes are not easily electrified, no alternative cement chemistry can be produced at a comparable scale and no natural source of un-carbonated calcium is available in the required quantities.

A widely deployed high-temperature electric process is the electric-arc furnace (EAF) used for recycling steel. This recycling occurs in two stages. First, scrap is melted and oxidized to remove dirt, carbon and phosphorus. Then, in a ladle furnace, sulfur is removed, and the steel is alloyed. In both steps, a flux is introduced to protect the steel from air, provide the required basicity, protect the lining and the graphite electrodes and increase energy efficiency. This flux is made from limestone–dolomite in a decarbonation process with the same process emissions as Portland cement^14^.

A widely available high-volume source of decarbonated calcium is found in the hydrated cement paste embedded in used concrete. It has been known for a long time that it is possible, in principle, to reclinker hydrated cement paste (HCP)^15^. However, there are several challenges to applying this process in a conventional kiln when a large fraction of hydrated cement paste is used. The presence of sulfates (added as set retardant during production) increases the belite content of the reclinkered cement at the expense of highly reactive alite^16^. Furthermore, sulfates are volatile and will condense in cooler parts of the kiln system, causing operational difficulties^17^. Nonetheless, there exist commercial offerings of cement made with partly replaced raw meal (such as Holcim GeoCycle). Separately, cement recovered from used concrete can contain a high fraction of chlorides that, if retained, would exclude the use of reclinkered cement in reinforced applications^18^.

Recovered cement paste (RCP) is not commercially available at scale at present. Separating paste from the aggregates in concrete has been a niche but active area of research and development^19,20^, driven not by the prospect of using the cement paste but by the possibility of recovering high-quality aggregates that have much higher commercial value than those produced from crushing concrete demolition waste^21^. The value of the improved recovered aggregates is not at present high enough to cover the extra cost of processing, so RCP is currently landfilled. However, the know-how and the technologies required to produce RCP at scale exist^22^. Recently, interest in RCP has grown because of interest in using it in a carbon mineralization process^23,24^, and start-up companies have been launched to sell specialist grinding equipment^25^. Thus, a nascent market for RCP now exists.

Here we report an innovation that arises from the combination of the above observations: electric cement recycling at scale. In the process shown in Fig. 3e, and described in the patent application^26^ for Cambridge Electric Cement (CEC), the emitting lime flux used in steel recycling is replaced by recovered cement paste that has already been decarbonated so will release no further process emissions, although it may require small adjustment with lime. The paste is reclinkered as it is fluxed into a slag. The higher temperature of the EAF (compared with cement kilns) both maintains as gas the sulfates and chlorides that held back earlier reclinkering trials and favours the production of alite over belite. The slag is cooled and ground to produce a conventional Portland clinker in an all-electric process, which, with a decarbonized grid, has neither process nor combustion emissions. This approach will in parallel reduce the emissions of steel recycling by reducing the need for flux production. Both steel recycling slags can be made cementitious by this approach, with the ladle slags being closer to cement composition. However, in this paper, we focus on the EAF (oxidizing) slags as their volume is larger.

In the context of steel recycling, fluxes are the minerals added to the steel and slag is the resulting viscous layer floating on top of the molten steel. In this paper, we aim to demonstrate that with the right composition of flux based on hydrated or recovered cement paste, the slag, when cooled rapidly, becomes clinker, the artificial mineral that, after grinding and blending, can be made into cement.

To evaluate the proposed new process route, 28 slags were produced from flux derived from cement paste both prepared for this purpose and recovered from demolition waste. Lime, alumina and silica were added to the fluxes. The composition of a selection of these slags is given in Table 1, with the full set specified in the Supplementary Information. The slags were processed in induction furnaces over clean steel with various crucibles and oxidizing conditions. In an industrial EAF, oxidation and reduction occur in a controlled sequence: these effects are tested separately here. The slags were air-cooled: at this scale, cooling rates of 10–20 K s^−1^ at least are achieved, which is fast enough to stabilize alite. The slags were then ground and characterized. Some of the slags with compositions matching conventional clinker listed in Table 2 were blended and used to make mortar bars.

**Table 1:** Main mass per cent oxide composition of selected slags produced in this study obtained by XRF

ID	Description	LOI	Na_2_O_eq_	MgO	Al_2_O_3_	SiO_2_	SO_3_	CaO	Fe_2_O_3_
GC80-1a	High alite	−2.63	0	1.2	4.7	20.8	2.5	64.0	5.5
GC80-2b	Medium alite	−2.13	0	1.1	5.3	22.2	2.1	64.7	3.5
AL250-5	High C_2_AS	−0.23	0.2	0.9	31.2	21.6	0.1	39.5	1.8
AL250-6	C_2_AS–C_2_S	−0.49	0.3	2.2	12.9	24.1	0.4	51.7	3.3
AL250-78:22	High C_3_A	−1.51	0.1	0.8	16.6	12.6	0.7	53.4	12.7
GC80-5e	High belite	−1.95	0.2	1.3	7.8	21.6	1.6	59.4	5.4
AL250-80:20	Amorphous	−1.17	0.2	0.7	29.4	12.0	0.3	45.7	8.7

Supplementary Table 1 has the complete dataset. A negative loss on ignition (LOI) indicates a gain in ignition. Gain in ignition is normal for slags as the oxidation state of the iron present increases; clinker typically has a loss in ignition.

**Table 2:** Phase mass per cent composition of selected slags produced in this study obtained by X-ray diffraction Rietveld analysis

ID	Description	C_3_S	C_2_S	C_3_A	C_4_AF	CaO	C_2_AS	C	Fe	Others
GC80-1a	High alite	60.9	14.8	8.6	0.7	0	4.6	5.0	2.4	3.1
GC80-2b	Medium alite	53.9	24.7	10.1	0.8	0.2	3.7	2.7	1.5	2.5
AL250-5	High C_2_AS	0	1.4	0	5.5	0	87.2	0	0	6.0
AL250-6	Medium C_2_AS	0	58.2	2.3	2.1	0	27.0	0	0	10.5
AL250-78:22	High C_3_A	10.3	33.2	23.0	23.6	0.9	0.4	0	0	8.7
GC80-5e	High belite	24.0	52.5	14.1	3.9	0.2	1.5	0.9	1.1	1.9

Supplementary Table 2 has the complete dataset.

**Fig. 1:**
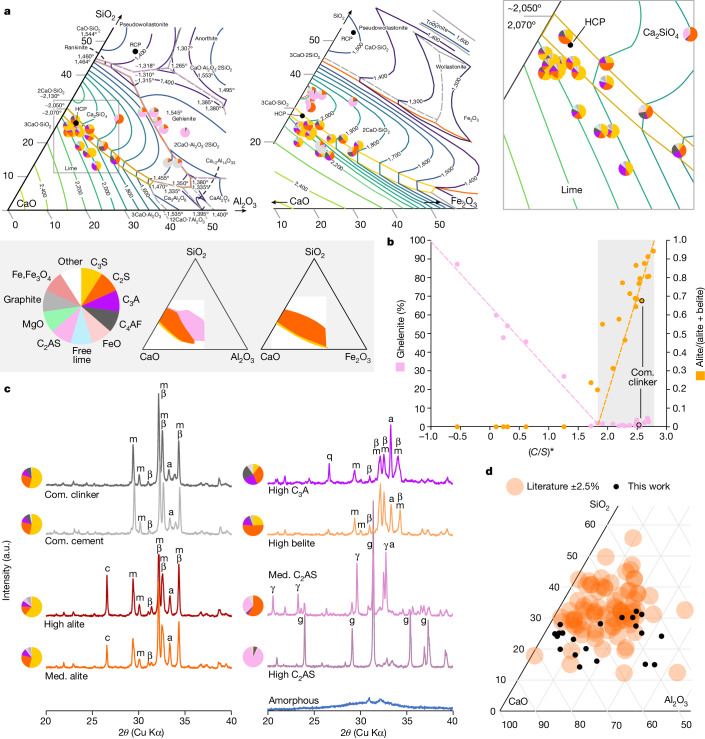
Chemical analysis of the clinkers produced over molten steel. **a**, Ternary diagram pair presenting the oxide composition of the slags studied in the SiO_2_–CaO–Al_2_O_3_ and SiO_2_–CaO–Fe_2_O_3_ systems measured using XRF. Every oxide composition was analysed by X-ray diffraction, and the resulting crystallographic composition is shown as a pie chart. Right, a detail of the SiO_2_-CaO-Al_2_O_3_ ternary diagram on the left. **b**, Percentage of gehlenite in the slag and the fraction of alite over total alite and belite in the tested systems both as a function of (*C*/*S*)*, the available lime-to-silica ratio for the formation of silicate phases. The method for calculating (*C*/*S*)* is given in the Methods. The grey shaded region represents the range of *C*/*S* for which both Alite and Belite can form. **c**, Diffractograms and phase compositions of selected slags produced in this work. γ, C_2_S-γ; β, C_2_S-β; m, C_3_S-monoclinic; g, ghelenite; a, C_3_A cubic; c, graphite; and q, quartz. **d**, Comparison between the tested slags and compositions reported in the literature. The literature used to create this figure is given in Supplementary Table 3. Med., medium; Com., commercial; a.u., arbitrary units.

The diffractograms of selected slags are shown in Fig. 1. The diffractograms of all slags close to from the alite-forming zone (Fig. 1a, yellow) have the main peaks of cementitious phases typical of Portland cement: alite (C_3_S), belite (C_2_S) as well as C_4_AF, and tricalcium aluminate (C_3_A). In general, the phases present are those predicted in the thermodynamic CaO–SiO_2_–Al_2_O_3_–Fe_2_O_3_ system (Fig. 1a). Rietvield refinement indicates that alite and belite together account for more than 70% of the products in most of the slags made in the zone in which commercial cement kilns operate (indicated by the narrow yellow region in Fig. 1a in which Al_2_O_3_ is below 6%). For reference, Portland cement should normatively contain 66.7% by mass of alite and belite^27^. Using this criterion, our process route can make Portland clinkers over a fairly wide zone. In the belite-forming zone (Fig. 1a, orange region), ghelenite is also sometimes observed. Ghelenite (C_2_AS) is the dominant species in the ghelenite zone (Fig. 1a, pink region). The effective lime-to-silica ratio (Methods) is an excellent predictor of the silicate phases that will form (Fig. 1b).

The X-ray fluorescence (XRF) analysis of selected slags is provided in Table 1 (for full results, see Supplementary Table 1). The oxide composition of the resulting slags does not differ markedly when using carbon or magnesium crucibles (Supplementary Fig. 1) indicating that over clean steel, there is no significant exchange of species between the slag and the melt. Early experiments performed with an aluminium oxide lining exhibited significant aluminium leaching (Supplementary Fig. 1) and were therefore abandoned. The contamination inherent to steel scrap used in commercial operations would introduce silica and alumina to the slag: the same final compositions would then be reached by adding more lime (CaO) to the flux.

RCP contains a higher proportion of SiO_2_ than HCP, almost certainly derived from aggregates still attached to the paste (Fig. 1a). Assuming that feedstock cement has the composition of the CEM I we used as a control, the RCP would contain 52% HCP and 48% impurities. Once corrected by adding lime to have the right balance of calcium and silica (Table 3), it reclinkers as well as pure hydrated paste.

**Table 3:** Raw meal compositions for reclinkering on molten steel

		Flux composition						
ID		C	S	A	F	M	N	$\bar{{\bf{S}}}$
MO2-1	HCP	66.7	20.1	5.0	3.6	1.0	0.4	2.4
MO2-3	HCP + lime + sand	67.5	23.7	3.3	2.3	0.9	0.4	1.5
MO2-4	RCP + lime + clay	67.8	22.0	5.2	1.8	1.1	0.7	0.7
MO2-5	HCP + clay	65.3	20.8	5.8	3.5	1.0	0.4	2.3
MO2-6a	HCP + clay	64.6	21.1	6.2	3.5	1.0	0.4	2.3
MO2-6b	HCP + clay	65.7	20.6	5.6	3.6	1.0	0.4	2.4
MO2-7	HCP Cl	63.1	19.3	5.0	3.6	0.8	2.2	2.7
GC80-1a	HCP	66.7	20.1	5.0	3.6	1.0	0.4	2.4
GC80-1b	HCP + clay	65.0	21.0	6.0	3.5	1.0	0.4	2.3
GC80-2b	EAFSc + lime	44.8	9.8	10.0	23.7	4.0	0.1	0.1
GC80-3a	Lime + clay + sand	68.8	19.2	10.1	0.3	0.7	0.4	0.1
GC80-3b	Lime + clay + sand + cor.	67.5	13.6	17.2	0.3	0.6	0.4	0.1
GC80-3c	Lime + clay + sand	68.0	17.4	12.6	0.4	0.7	0.5	0.1
GC80-4a	HCP	66.7	20.1	5.0	3.6	1.0	0.4	2.4
GC80-5a	HCP Cl	63.1	19.3	5.0	3.6	0.8	2.2	2.7
GC80-5b	EAFSc + HCP + lime	54.6	12.7	7.9	16.0	2.9	0.2	0.8
GC80-5c	EAFSm + HCP + lime	51.0	11.3	7.7	15.3	5.3	0.1	0.7
GC80-5d	RCP + lime + clay	67.8	22.0	5.2	1.8	1.1	0.7	0.7
GC80-5e	HCP + clay	61.6	22.7	7.9	3.4	1.0	0.5	2.2
AL250-1	RCP + lime + add.	55.7	29.3	8.9	2.3	1.2	0.8	1.0
AL250-2	HCP + lime + sand + add.	60.2	30.0	6.0	1.4	0.7	0.4	0.9
AL250-3	HCP + lime + sand + add.	62.1	26.3	4.4	3.1	0.9	0.4	2.1
AL250-4	HCP + lime + sand	69.0	22.4	3.1	2.2	0.9	0.4	1.4
AL250-5	HCP + lime + sand	69.0	22.4	3.1	2.2	0.9	0.4	1.4
AL250-6	HCP + lime + sand	69.0	22.4	3.1	2.2	0.9	0.4	1.4
AL250-80:20	HCP + lime	73.9	15.7	3.9	2.8	1.0	0.3	1.9
AL250-79:21	HCP + lime	74.2	15.5	3.8	2.8	1.0	0.3	1.8
AL250-78:22	HCP + lime	74.6	15.3	3.8	2.7	1.0	0.3	1.8

All values are in % mass. HCP, hydrated cement paste; RCP, recovered cement paste; Clay, Al_2_O_3_-rich kaolin clay; EAFSc and EAFSm are two different EAF slags; Add., reducing additives such as metallic Al and ferrosilicon; Cor., corundum. HCP Cl, paste saturated with chloride. The data under flux composition give the mass composition of the effective final flux. Cement chemistry notation is used. C, CaO; S, SiO_2_; A, Al_2_O_3_; F, Fe_2_O_3_; M, MgO; N, Na_2_O_eq_; and $\bar{{\mathsf{S}}}$, SO_3_.

The metallurgical function of EAF oxidizing slags is to allow the dephosphorization of steel while limiting sulfur in the melt. The slag compositions we tested overlap with those reported in the literature (Fig. 1d) and have the appropriate chemistry. To verify their suitability for EAF operations, slags of typical composition were used, and the resulting steel composition was tested but using a considerably larger flux-to-steel ratio than would be practical in present-day furnace designs (roughly 9:30 compared with 1:30). Despite these unfavourable conditions, only very small amounts of sulfur transferred into the steel (Table 4). In steel-making, following further desulfurization, so far most alloys have a sulfur concentration of less than 0.05% (ref. ^28^), which can be achieved easily from the values reported in Table 4. This confirms that the basic slags produced from the proposed process can be used in EAFs to de-phosphorize steel.

**Table 4:** Optical emission spectroscopy of the steel produced for two selected melts (mass %)

MO2	C	Si	Mn	P	S	Cr	Mo	Ni	Al	N_2_
1	0.003	-	0.01	-	0.07	-	-	-	-	0.008
6b	0.002	-	-	-	0.08	-	-	-	-	0.007

The row labels are slag IDs taken from Table 3. The initial steel is pure iron within the detection threshold.

**Fig. 2:**
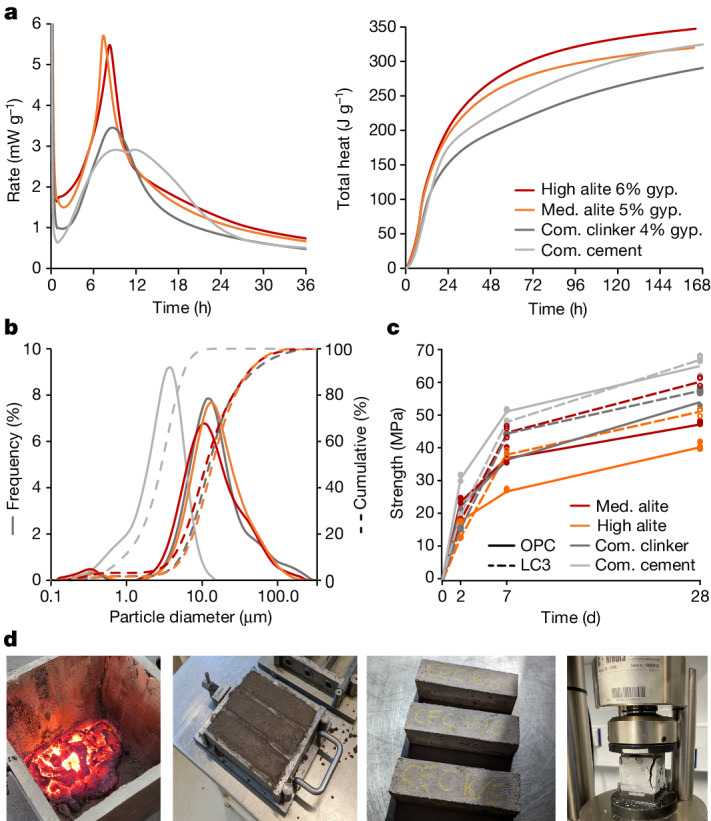
Performance analysis of a selection of clinkers produced with the new process. **a**, Instantaneous and cumulative heat released by high- and medium-alite cement and high-C_3_A cement produced with the new process, commercial clinker ground in the same conditions and commercial cement produced with the commercial clinker shown for reference. **b**, Cumulative and frequency plots of the particle size distributions of the cements used for the strength tests. **c**, Strength evolution of cements produced using the new process, both as pure cements and LC^3^ blends. **d**, Slag as poured from the furnace, fresh and hardened mortar bars; sample after compression failure. Med., medium; Com., commercial; gyp., gypsum.

The cement setting time was measured using calorimetry at 240–280 min (Fig. 2a, ±20 min after^29^, the middle point between the dormant period trough and the peak of the silicate hydration peak). Mortars made with the cements were fairly easy to place with small additions of superplasticizer required for the slags made in graphite crucibles (GC80) and for LC^3^ mixes. No early setting or flash setting was observed. No bleeding was observed (Fig. 2d, second from the left). On demoulding, the samples were inspected and no defects were found (Fig. 2d, third from the left). All cements, both pure and those blended with calcined clay and limestone, exhibited similar strength development to those made with commercial clinker despite being undersulfated (Supplementary Fig. 2), and containing a fraction of contaminants probably introduced when the crucible was scraped during tapping (Fig. 1c). The relationship between strength development and heat release (Fig. 2a,c) for both our new clinker and a conventional commercial clinker was similar. Higher belite content is associated with lower early strength, whereas higher alite relates to higher early age strength (Fig. 2a and Supplementary Table 2). The improved performance of commercial cement (compared with that made from commercial clinker) is because of finer grinding^30^ (Fig. 2b). With commercial grinding equipment the clinker produced with the new process is likely to have the same grindability as commercial clinkers (Fig. 2b).

Together, these results demonstrate the production of Portland cement by reclinkering HCP or RCP on a bed of molten steel. The slags arising from the new flux have the right composition for their metallurgical function. The mineralogy of the output can be tuned as in any conventional kiln, and high-quality clinker can be produced.

**Fig. 3:**
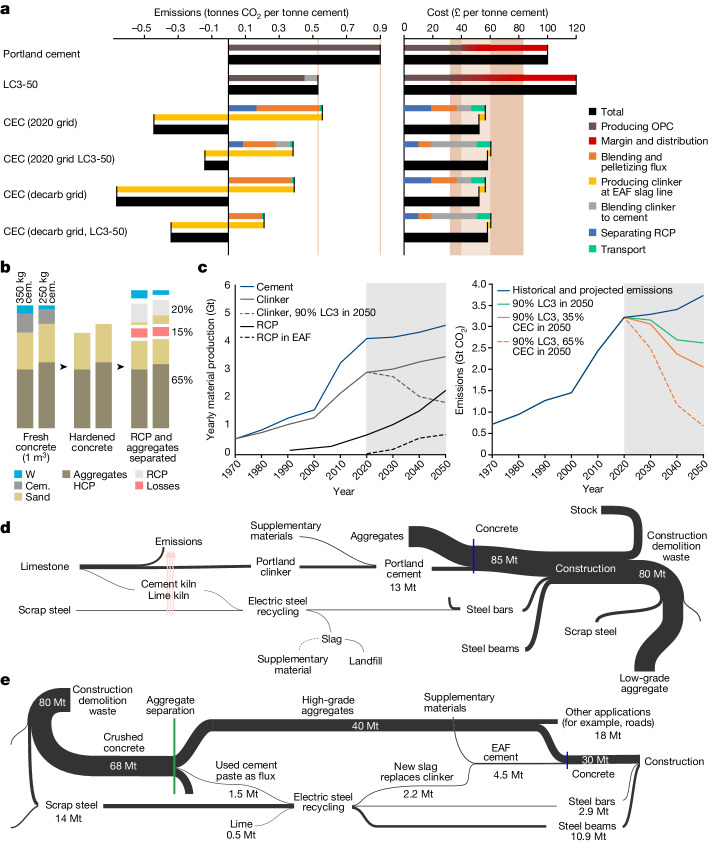
Environmental and economic analysis. LC^3^-50 is 50% clinker, 30% calcined clay, 15% limestone and 5% gypsum; CEC is the process described in this paper. **a**, Emissions are estimated based on global data, whereas costs are estimated for the UK. The four recipes for electric cement (CEC) use global average or future zero emissions electricity, with or without blending with calcined clay and are compared with ordinary Portland cement and LC^3^-50. Full details of the sources and calculation of these estimates are provided in the Supplementary Information. **b**, Representation of the range of concrete compositions and the outcomes of separation (W is water). **c**, Historical and projected cement and clinker production worldwide, implied RCP availability assuming a 50-year lifetime for buildings (left); global cement-related emissions under a range of scenarios (right); the potential supply of RCP is greater than that which could be used in EAFs but production could be increased if new dedicated EAFs are built to produce cement. **d**, The current material flows and industrial operations for the production of bulk construction materials. **e**, The material flows anticipated if the process described in this paper is deployed at scale in the UK and all arising steel scrap is recycled domestically. cem., cement; decarb, decarbonized.

Figure 3a confirms the emissions saving from electric cement recycling and anticipates production costs comparable with those of present-day cement that are dominated by the price of fuel and SCMs^31^. All processes are electrified, and both the extra costs and total power requirements of the new process are dominated by heating old concrete before crushing. This is currently required to separate a clean stream of RCP but will be optimized in future. With a decarbonized grid, the only emissions of co-production arise from the small remaining requirement for lime flux, and these would be allocated between the two materials by negotiation. The amount of lime flux required in EAFs depends on the steel scrap feedstock and the furnace design. Clean steel requires less flux to bind residue from the steel, but more to create enough slag to cover the electrodes. All such flux could be replaced with RCP to produce cement. By contrast, although less clean steel requires less flux to cover the electrodes, more is needed to bind with unwanted silica to ensure high slag basicity required for steel quality. In this case, adding RCP to the flux would not reduce lime use but would still produce additional mass of clinker without increasing the emissions of steel recycling. Therefore, Fig. 3a distinguishes the emissions savings from a typical reduction in lime use and those from avoiding conventional cement production. The fact that operating expenses are much greater than capital or labour costs, suggests that the market viability demonstrated here for the UK will translate to other countries.

The overall supply chain reconfiguration is extensive (compare Fig. 3d,e), but every change can be economically viable. Figure 3d draws on current estimates of mass flows in the UK: steel demand is around 15 Mt yr^−1^, mainly from imports, leading to around 10 Mt yr^−1^ of scrap, most of which is currently exported^32^; cement demand is around 13 Mt yr^−1^, requiring clinker production of around 9 Mt yr^−1^ (ref. ^33^). Total construction and demolition waste is around 68 Mt yr^−1^ (refs. ^34,35^), most of which is concrete. A conservative estimate is that 4–4.5 Mt of RCP could be produced with a 60% collection rate. The data in Fig. 3e assume that scrap steel volumes approach annual demand to reach 14 Mt yr^−1^ and that all UK steel scrap is recycled domestically, following the expansion of EAF capacity. This defines the total capacity for electric cement production. In this study, experiments used 5–30% of the steel mass as the input flux mass. EAFs can operate now with up to 20–30% slag. Although this could be increased, higher values are at present considered undesirable as slag is a waste by-product now, so the figure assumes a slag-to-steel ratio of 1:7, or 14%.

## Discussion

All processes, norms, practices and habits in the construction industry are centred around Portland cement. Therefore, it would be difficult for any new material to replace it, unless it had considerable advantages. As Portland cement is made from the most abundant elements in the crust of Earth, it is unlikely that any potential substitute could be cheaper or available in equivalent volume. Despite its requirement for high temperatures, the present production of Portland cement is already energy-efficient, exploiting molten iron and aluminium oxide phases to transport ions. Therefore, the only practical avenues for decarbonizing cement production depend on altering the range of allowable compositions, using new SCMs or changing the process by which cement is produced. Recycling Portland cement as proposed here saves the process emissions and the bulk of the energy required for production^36^.

The proposed industrial symbiosis needs careful development: both steel-making and clinkering processes require careful chemical tuning but as steel is more expensive, its requirements would dominate decision-making. Substituting RCP for lime flux should make no marked change to the steel quality or the air pollution created during the EAF process, so the existing regimes of exhaust scrubbing should remain appropriate. In co-production with steel, cement would have a higher iron content because of rheological requirements for slags. However, our experiments show that the cement composition can be tuned with additions of lime to target-optimized blends. The cement produced using this new process can have a higher alite fraction than previously published results^15^ or commercial clinker (Fig. 1b).

Figure 3e suggests that about 2.2 Mt of the new clinker could be produced in the UK annually in co-production with steel. If this is blended with calcined clay, it could make about 4.5 Mt of LC^3^-50 cement. This could meet the final demand when combined with the strategies of material efficiency. In particular, avoiding overdesign^9^, extending life and increasing the use^10^ could deliver the services with less than half of total cement production. However, the total production of the electrically recycled cement in the UK will not be limited by RCP availability. EAF operators could choose to produce more slag than what is produced now, which might also increase the quality of steel production. The increase in costs would be offset by new revenue from the recycled cement. If EAF operators find this commercially attractive and double the amount of slag produced with each batch of steel, the overall output of this process in the UK could be as much as 10 Mt.

Figure 3c expands this analysis to look at the global potential scale of the new process. In the present work, we have assumed that electrically recycled cement will initially be made in co-production with recycled steel, to minimize new capital investments. If global EAF capacity expands as anticipated and all resulting slag is processed into LC^3^ cements, this would lead to around 1.4 Gt of electric cement and 2 Gt of CO_2_ abatement. However, if additional dedicated EAFs are installed and used solely for the electrical production of cement over a constant volume of steel, we estimate that global production of the new cement could be as much as 2.4 Gt leading to emissions abatement of 3 Gt compared with a scenario with no abatement. This represents an 80% abatement of sectoral emissions otherwise expected in 2050. The total potential is constrained not only by EAF capacity but also by the quality of RCP separation and the contamination of steel scrap.

## Conclusion

This study demonstrates that using existing industrial-scale equipment it is possible to recycle Portland cement into Portland cement in an all-electric process. The process can be operated as co-production with steel recycling or for the exclusive production of cement using an EAF with a small untapped pool of molten steel. The process is fundamentally a material substitution within existing processes, equipment and standards, and so it could scale rapidly. It creates the first zero-emissions alternative to existing cement production, to our knowledge, which accounts for 7.5% of the present anthropogenic emissions.

## Methods

We have prepared cement pastes and mortars for demonstrating the process as well as blending cements for characterization and to make mortar.

### Materials

To prepare HCP, cement provided by Tarmac (CEM I 52.5N) was used with a water-to-cement ratio of 0.6 at room temperature. The oxide composition of the original cement is given in Extended Data Table 1, which also specifies the oxide composition of the other additions used to create the fluxes in these trials. The cement was kept in sealed containers and cured for 6 months to ensure it was almost fully hydrated. The blocks of cement pastes were then fired at 500 °C in a muffle furnace. The final loss on ignition was 13.7%. Recovered paste was obtained from the dust collector of the concrete processing plant of Day Aggregates. It was not subject to any treatment.

The raw meal flux was prepared using homogeneously mixed powders crushed to less than 5 mm in size.

Several steel sources were used: high-purity steel (electrolytic iron flake from Willian Rowland, lot no. 16M-134), steel ingots and steel balls (composition measured by optical emission spectroscopy is given in Extended Data Table 2).

The ground clinkers were blended with calcium sulfate dihydrate (gypsum) in powder form (VWR Chemicals, AnalaR NORMAPUR) to make cements. The amount of gypsum was adjusted to allow measurement of the effect of gypsum addition on early age reactivity (Fig. 2a and Supplementary Fig. 2). The mortars were prepared following EN 196-1, using standard sand (Société nouvelle du littoral) at a water-to-binder ratio of 0.5. Limestone calcined clay cements (LC^3^) were prepared by blending clinker (50%) with 30% metakaolin (supplied by Imerys), 15% ground limestone and 5% calcium sulfate dihydrate. The mix proportions are given in Extended Data Table 3. The addition of some superplasticizers (Masterglenium 51, Master Builders) was necessary to ensure workability for some mixes, particularly with the LC^3^ blends.

### Methods

Three furnaces of different sizes and operating conditions were used.

A small-size magnesium oxide crucible (MO2) of 100 mm height and 50 mm diameter, under inert conditions (flushed with argon) was used with 500 g high-purity steel and 100 g powder of raw meal flux. The flux was loaded on top of the steel, which was heated up to 1,650–1,750 °C and held for 15 min. The slag was stirred once using a steel rod during the heating cycle and was tapped out on a 20-mm thick steel plate. The slag samples were slightly oxidized during the mixing and tapping out process.

A medium-size graphite crucible (GC80) of dimensions 300 mm height and 100 mm diameter loaded with 12 kg iron balls and 2 kg raw meal powder was heated in reducing conditions (due to the leaching of carbon from the crucible), in an induction furnace powered by three-phase 50 Hz electricity, generating single phase output power of 112 kW, 1,200/600 V and 220/440 A at 2–3.3 kHz. The furnace is equipped with a 750-l capacity water tank for cooling, operated between 2.8 bar and 6.2 bar pressure, providing IP54 degree of protection. The flux was loaded on top of the steel and heated to 1,650–1,750 °C. During the process, it was stirred with long steel rods. After holding at this temperature for 15 min, the slag was tapped off using steel rods to scrape it into a steel box. The slag was spread on the box to ensure fast cooling. After the slag was tapped off, new flux was added, repeating the entire process. Typically, three to five different slags were produced from each of the melts.

A large-size induction furnace with alumina lining (AL250) from Capital Refractories with a Coral GR9 lining made of 80–90% aluminium oxide and 10–20% magnesium oxide was used in mildly oxidizing conditions. The furnace is powered by a three-phase 295 kVA, 415 V, 50 Hz power supply, generating an output of 250 kW and 1,000 Hz. The AL250 had 700 mm height and 300 mm diameter and was loaded with 200 kg steel ingot and 10 kg raw meal powder. First, steel was allowed to melt and then flux was added on top. The furnace was heated up to 1,650–1,700 °C for about 30 min. The slag was tapped into a steel box for cooling.

After cooling, the slags were crushed to less than 5 mm to allow for the removal of steel residuals using magnets. Furthermore, the slag from the small furnace (MO2) was ground in a planetary ring mill for 30–60 s, and the slags from the other two furnaces were ground in a ball mill with steel balls as charge. The ball mill was operated for 3 h at 16 rpm, with charge-to-feed-mass ratio of 1:7. The raw meal was prepared using different materials to cover a range of flux composition. Further details of raw meal blending are given in Table 3.

X-ray fluorescence (XRF) analyses were performed with a Rigaku ZSX Primus IV XRF spectrometer, using the quantitative Fluxana Raw calibration application. Lithium borate was used as the flux in the bead-making process. The sample-to-flux ratio was 1:10. All samples were fired in a muffle furnace before bead making. The heating profile was as follows: set point 900 °C, heating rate: 3 °C min^−1^, hold time: 5 h.

X-ray diffraction was performed with a Bruker D8 advance spectrometer with cobalt anode (Co Kα_1_ = 1.789 Å) operated at 35 kV and 35 mA. A cobalt source was preferred over copper to minimize the fluorescence from Fe–K edge emissions. The samples were front-loaded and the scan range was 5–70° 2*θ* with a step size of 0.02° 2*θ* and a time per step of 0.5 s. Each measurement took around 30 min. The Rietveld analysis to quantify the crystalline phase content was performed using Topas Academic v.7 software. The lattice parameters and crystallite size were allowed to refine, whereas the atomic positions were not refined. The amorphous content of the two highly amorphous samples was determined by the external standard method using corundum as the standard. Determining the amorphous content of other clinkers was difficult as a McCrone mill was not available, and it is crucial to mill the particle down to low fineness to ensure the amorphous content is measured appropriately^37^.

Optical emission spectroscopy measurements were performed with an Amtek Spectrolab S.

The mortars were mixed according to the provisions of EN 196-1. The mix proportions are given in Supplementary Table 3. The surface of the mortar was smoothed using a trowel, the mortars were covered with a plastic wrap and left to set for 24 h. At 24 h, the mortars were demoulded and put to cure under water in a room of temperature 23 ± 2 °C.

For strength testing, the samples were taken out of the water and wiped. The bars were cut using a diamond saw into 40 mm cubes. The cubes were loaded in compression, as per EN 196-1, with the casting surface on the side and a loading rate of 2,400 N s^−1^. The peak load (*L*) and the area of the cube (*A*) were recorded, and the compressive strength (*F*_c_) on days 2, 7 and 28 was reported as

1 $${F}_{{\rm{c}}}=\frac{L}{A}$$

(*C*/*S*)* is the ratio relevant for the formation of calcium silicates. It is calculated from the CaO, SiO_2_, Al_2_O_3_ and Fe_2_O_3_ content from XRF, as well as FeO and metallic iron (Fe) and the free lime content determined from X-ray diffraction (XRD). The total Fe_2_O_3_ content in slag is determined from

2 $${{\rm{Fe}}}_{2}{{\rm{O}}}_{3}={{\rm{Fe}}}_{2}{{\rm{O}}}_{3}^{{\rm{XRF}}}-1.11\times {{\rm{FeO}}}^{{\rm{XRD}}}-1.43\times {{\rm{Fe}}}^{{\rm{XRD}}}$$

All the Fe_2_O_3_ is then assumed to form C_4_AF. The CaO required for the formation of C_4_AF is

3 $${{\rm{CaO}}}^{{{\rm{C}}}_{4}{\rm{AF}}}=1.4\times {{\rm{Fe}}}_{2}{{\rm{O}}}_{3}$$

And the CaO required to form C_3_A from the residual Al_2_O_3_ is

4 $${{\rm{CaO}}}^{{{\rm{C}}}_{3}{\rm{A}}}=1.645\times ({{\rm{Al}}}_{2}{{\rm{O}}}_{3}-0.64\times {{\rm{Fe}}}_{2}{{\rm{O}}}_{3})$$

Then (*C*/*S*)* is

5 $${\left(\frac{C}{S}\right)}^{* }=\frac{{{\rm{CaO}}}^{{\rm{XRF}}}-{{\rm{CaO}}}^{{{\rm{C}}}_{4}{\rm{AF}}}-{{\rm{CaO}}}^{{{\rm{C}}}_{3}{\rm{A}}}-{{\rm{CaO}}}^{{\rm{free,XRD}}}}{{{\rm{SiO}}}_{2}^{{\rm{XRF}}}}$$

Negative values of (*C*/*S*)* indicate that no alite or belite can form. However, the amount of lime, alumina and silica in the system determines the amount of ghelenite formed.

## Online content

Any methods, additional references, Nature Portfolio reporting summaries, source data, extended data, supplementary information, acknowledgements, peer review information; details of author contributions and competing interests; and statements of data and code availability are available at 10.1038/s41586-024-07338-8.

### Supplementary information

Supplementary Information

## Figures and Tables

**Extended Data Table 1:**
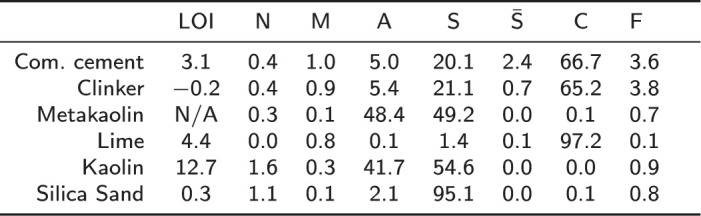
Main mass percent oxide composition for the materials used in this study

“Com. cement” is the commercial cement, “Clinker” indicates commercial clinker. Negative LOI indicates a gain in ignition. Gain in ignition is normal for slags as the oxidation state of the iron present increases; typically, clinker has a loss in ignition. Cement chemistry notation is used. C: CaO, S: SiO_2_, A: Al_2_O_3_, F: Fe_2_O_3_, M:MgO, N:Na_2_O_eq_, $\bar{{\mathsf{S}}}$:SO_3_.

**Extended Data Table 2:**
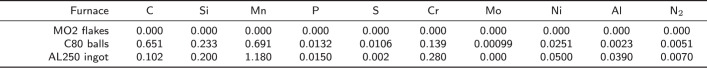
Optical Emission Spectroscopy of the steel used in the different furnaces (mass %)

The remainder is Fe.

**Extended Data Table 3:**
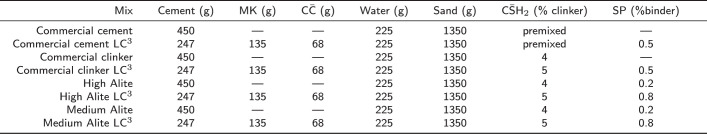
Mix proportion of mortar mixes

“MK” is metakaolin, “Clinker” indicates commercial clinker, C$\bar{{\mathsf{C}}}$ is limestone powder, C$\bar{{\mathsf{S}}}$H_2_ is gypsum, SP is superplasticiser.

## Data Availability

All data from the experiments reported in this paper are contained in the tables of the main paper and Supplementary Information.
