# Supplementary material for: Electric recycling of Portland cement at scale
Source: Nature. 2024 May 22;629(8014):1055–61. doi: 10.1038/s41586-024-07338-8 (PMC11136652; doi:10.1038/s41586-024-07338-8)
Supplement: Supplementary file 1 — Supplementary Information [file 41586_2024_7338_MOESM1_ESM.pdf]

nature portfolio

https://doi.org/10.1038/s41586-024-07338-8

Supplementary information

Electric recycling of Portland cement at scale

In the format provided by the authors and unedited

Electric recycling of Portland cement at scale

Cyrille F Dunant, Shiju Joseph, Rohit Prajapati, Julian M Allwood

Supplementary Information

Contents:

Material identification 2
Literature survey of EAF slag compositions 4
Experimental notes 13
Emissions and economic analysis of electric cement recycling in the UK 17
Analysis of potential global scale of electric cement recycling 25
Additional references 27

Material identification

The oxide composition of all slags and raw materials were determined as described in the materials and methods. Supplementary Table 1 gives the resulting oxide composition. A value for Chloride is reported, but it is only indicative due to the calibration of the XRF equipment.

Melt #	Melt ID	XRF										
		CaO	SiO2	Al2O3	Fe2O3	MgO	MnO	Cr2O3	SO3	Na2O	K2O	LOI
**Melts reported in the paper**												
1	MO2-1	64.90	20.40	3.10	7.40	1.00	0.10	0.00	0.60	0.00	0.00	-0.89
2	MO2-3	66.60	23.30	2.60	4.40	1.90	0.00	0.00	0.70	0.00	0.00	-0.50
3	MO2-4	65.90	21.80	3.40	4.70	3.10	0.00	0.00	0.24	0.02	0.02	-0.40
4	MO2-5	60.84	23.44	3.06	8.36	1.82	0.05	0.02	0.64	0.04	0.09	-0.45
5	MO2-6a	62.71	21.15	4.87	7.04	0.91	0.06	0.03	1.37	0.05	0.11	-1.85
6	MO2-6b	57.51	19.57	3.51	16.22	0.82	0.05	0.02	0.75	0.06	0.11	-1.78
7	MO2-7	60.13	19.51	2.97	13.50	0.61	0.04	0.02	0.91	0.52	0.07	-2.04
8	GC80-1a	64.00	20.80	4.70	5.50	1.20	0.10	0.00	2.50	0.00	0.00	-2.63
9	GC80-1b	64.70	22.20	5.30	3.50	1.10	0.10	0.00	2.10	0.00	0.00	-2.13
10	GC80-2b	46.00	13.00	10.70	15.90	4.50	4.90	2.10	0.20	0.10	0.00	-2.63
11	GC80-3a	63.24	20.66	9.44	3.74	0.29	0.10	0.02	0.26	0.02	0.10	-0.56
12	GC80-3b	67.94	12.93	15.78	0.35	0.28	0.04	0.01	0.02	0.02	0.03	0.20
13	GC80-3c	68.04	16.51	12.59	0.35	0.30	0.03	0.00	0.01	0.01	0.02	-0.35
14	GC80-4a	63.66	20.06	4.58	7.26	1.07	0.17	0.03	2.21	0.02	0.03	-2.18
15	GC80-5a	52.90	17.22	3.98	19.29	0.82	0.38	0.06	1.99	0.67	0.06	-4.98
16	GC80-5b	62.71	16.66	7.40	4.82	2.94	1.71	0.45	0.80	0.07	0.05	-1.39
17	GC80-5c	52.91	12.87	8.66	9.96	5.81	3.66	1.82	0.59	0.04	0.04	-1.76
18	GC80-5d	63.19	20.64	5.94	4.79	0.90	0.14	0.03	1.08	0.18	0.08	-2.75
19	GC80-5e	59.43	21.63	7.80	5.42	1.31	0.29	0.06	1.60	0.09	0.20	-1.95
20	AL250-1	38.70	23.30	18.20	10.20	2.10	4.60	0.40	0.10	0.22	0.30	
21	AL250-2	43.60	23.90	23.10	1.70	2.70	2.00	0.10	0.20	0.10	0.20	-0.39
22	AL250-3	44.20	25.00	17.50	3.40	2.80	5.10	0.30	0.20	0.00	0.30	-0.72
23	AL250-4	42.50	21.20	24.40	1.80	7.70	1.90	0.10	0.30	0.10	0.30	-0.39
24	AL250-5	39.50	21.60	31.20	1.80	0.90	2.60	0.10	0.10	0.10	0.20	-0.23
25	AL250-6	51.70	24.10	12.90	3.30	2.20	2.30	0.10	0.40	0.10	0.30	-0.49
26	AL250-80:20	45.65	12.09	29.35	8.70	0.70	0.90	>0.08	0.30	0.10	0.15	-1.17
27	AL250-79:21	42.95	12.05	31.70	7.43	1.00	>0.1	>0.08	0.10	0.06	0.12	-1.07
28	AL250-78:22	53.36	12.60	16.60	12.65	0.80	>0.1	>0.08	0.66	0.06	0.09	-1.51
**Commercial comparisons**												
	Commercial clinker	65.18	21.05	5.43	3.79	0.89	0.09	0.02	0.66	0.21	0.26	-0.20
	Commercial cement	65.35	19.62	4.86	3.41	0.99	0.08	0.01	2.36	0.10	0.45	0.16
**Feedstock materials used in trials**												
	HCP	65.27	19.64	4.91	3.52	1.01	0.10	0.01	2.34	0.12	0.42	13.70
	HCP Cl	63.09	19.32	5.02	3.62	0.84	0.09	0.01	2.70	1.86	0.50	13.01
	EAFSm	35.94	8.85	9.85	22.12	7.60	7.22	4.36	0.07	0.02	0.02	-2.71
	EAFSc	19.86	13.45	14.36	34.13	5.36	5.74	3.25	0.09	0.17	0.03	-3.13
	Lime	95.79	1.43	0.10	0.14	0.81	0.03	0.01	0.13	0.01	0.03	4.42
	Sand	0.11	91.05	2.01	0.74	0.09	0.02	0.05	0.00	0.18	1.32	0.32
	Kaolin	0.02	52.10	39.79	0.83	0.27	0.01	0.00	0.00	0.09	2.05	12.69
	Metakaolin *	0.13	49.20	48.40	0.68	0.09	0.00	0.02	0.05	0.21	0.16	N/A
	RCP	30.38	48.51	7.42	4.39	1.64	0.11	0.14	1.76	0.85	1.10	23.35
	** data provided by KU Leuven*											

Supplementary Table 1: XRF of all the slags tested as well as the raw materials. The calibration of the XRF does not allow for accurate measure of chloride content.

The crystalline content of all the slags was analysed, following the protocol given in the materials and methods. The results of this analysis are reported in Supplementary Table

2. For most of the materials, the amorphous content was negligible. Except for a few samples (MO2-3 to MO2-7), there is an offset of around 2.65° 2θ due to the loss of alignment of the diffractometer. Nevertheless, apart from that offset, there was no difference in peak intensities.

Melt #	Melt ID	XRD																											
		C3S M3	C2S Beta	C3A cubic	C4AF Juoe	FeO	Spinel	C2S gamma	C2AS	MgO	Graphite	Lime	o-C3A	Alpha iron	C2F	Portlandite	Magnesioferrite	Chromite	MgO MnO	Mn3O4	Akermanite	Quartz	Merwinite	Fe2O3	bredigite	Calcite	Anhydrite	Gypsum	Amorphous
1	MO2-1	72.5	4.8	5.7	8.2	2.5	1.6	0.0	0.7	0.3		1.3		1.0		1.4													
2	MO2-3	63.5	16.6	3.9	4.6	0.9	0.8	2.5	1.0	2.8	0.0	0.4	0.0	0.4	0.0	2.5	0.0												
3	MO2-4	59.5	12.5	5.5	6.1	0.9	1.9	2.8	0.0	4.6	0.0	0.7	0.0	0.2	0.0	3.8	1.5												
4	MO2-5	35.6	33.0	3.8	13.6	2.2	0.4	8.0	0.4	1.5	0.0	0.5	0.0	1.0	0.0	0.0	0.0												
5	MO2-6a	49.7	25.9	8.4	8.3	0.7	1.5	1.5	1.2	0.8	0.0	0.3	0.0	1.1	0.0	0.4	0.3												
6	MO2-6b	38.2	22.6	1.7	11.2	2.6	1.4	5.4	0.5	0.7	0.0	0.8	0.0	2.5	10.8	0.2	1.3												
7	MO2-7	63.3	8.7	1.1	7.0	1.9	2.1	1.2	2.2	0.4	0.0	0.8	7.0	3.1	0.0	1.4	0.0												
8	GC80-1a	60.9	13.4	8.6	0.7	0.3	1.8	1.3	4.6	1.0	5.0	0.0	0.0	2.4	0.0	0.0	0.0												
9	GC80-1b	53.9	22.8	10.1	0.8	0.2	1.3	2.0	3.7	0.9	2.7	0.2	0.0	1.5	0.0	0.0	0.0												
10	GC80-2b	9.0	31.2	5.7	21.6	13.7	0.8	5.2	1.9	0.1	0.4	3.7	0.0	0.0	0.0	0.9	0.0	3.3	2.6										
11	GC80-3a	32.9	24.9	17.0	1.9	0.1	1.3	2.0	1.7		10.5	4.2		0.1		3.6													
12	GC80-3b	41.3	7.8	34.6	0.3	0.1	0.2	2.1	2.7		3.9	7.0		0.1															
13	GC80-3c	54.6	5.3	20.5	0.2	0.3	0.6	2.4	2.2		9.7	3.4		0.1		0.8													
14	GC80-4a	69.8	5.1	10.9	1.1	0.5	2.0	1.7	4.3		2.4	0.7		1.6															
15	GC80-5a	50.4	21.6	4.9	2.4	0.2	0.4	1.5	3.4	0.0	1.3	3.4	7.2	2.2	1.0														
16	GC80-5b	42.9	17.9	13.0	6.9	0.3	1.2	1.5	0.6	2.2	5.2	8.0	0.0	0.4	0.0														
17	GC80-5c	40.4	14.1	15.0	11.6	3.7	0.3	2.1	0.8	2.1	0.1	6.3		0.7				1.4	1.5										
18	GC80-5d	60.2	15.8	9.3	1.1	0.2	2.9	2.0	0.9	0.5	0.5	4.4	0.0	0.6	0.0	1.7													
19	GC80-5e	23.9	50.9	14.1	3.9	0.3	0.9	1.6	1.5	0.7	0.9	0.2	0.0	1.1	0.0														
20	AL250-1		4.4	0.8	1.0	5.0	0.9	9.0	54.1						7.5			1.0		3.4	7.1	0.7	5.3						
21	AL250-2		8.6			2.7	2.2	5.7	47.8	3.4					1.9						10.9		10.8	6.0					
22	AL250-3		2.9			6.0	3.5	20.6	45.6	7.0										0.3					14.2				
23	AL250-4		9.7		0.5	3.6	0.7	9.6	59.8	1.2					1.0				1.3	1.1	3.4				8.3				
24	AL250-5					2.8	1.5	1.4	87.2						5.5			0.5		1.2									
25	AL250-6		10.8	2.3	1.5	2.8	0.8	47.4	27.0						0.6			0.5	2.0		4.5								
26	AL250-80:20	3.3	4.0	0.9	2.7	0.4	0.0	1.5	0.0	0.0	0.0	0.0	0.0	0.0	0.0						3.1								84.0
27	AL250-79:21	4.6	11.4	0.5	1.0	0.6	0.0	2.7	11.5	0.0	0.0	0.0	0.0	0.0	1.0						5.7								61.0
28	AL250-78:22	10.3	27.8	23.0	23.6	2.5	1.6	5.4	0.4			0.9										4.6							
	Commercial clinker	54.1	22.8	9.4	9.2	0.0	0.2	3.2	0.5	0.1	0.0	0.6	0.0		0.0														
	Commercial cement	53.2	22.7	9.1	8.8	0.0	0.2	2.8	0.2	0.1		0.7				0.4										0.6	0.3	1.0	

Supplementary Table 2: XRD phase composition from Rietveldt analysis.

Determination of the amorphous content in the other slags/clinker is difficult as the amorphous content is lower than 20% so its presence cannot be visually identified. Quantitative determination of the amorphous content using either the internal or external standard method requires a McCrone mill which was not available. For example, Snellings et al. (2014) showed clinker and alite having mean particle size of around 35µm could have amorphous content of around 20%, while when the same clinkers are ground down to around 3-5 µm, the amorphous content decreases to close to 0%. Therefore, we deduce that the clinkers produced in the experiments reported in this paper have an amorphous content no more than 20%, but this is likely to be a substantial overestimate based on the performance and concordance between XRF and XRD compositions.

Two slags (AL250-80:20 and AL250-79:21) have high amorphous content as shown by the diffractogram. To determine the amorphous content in these two slags, the external standard method approach was used (Jansen et al., 2011) with corundum (Al_2O_3) used as an external standard. In using this method, it is important to find the mass attenuation coefficient (MAC), which was calculated using the XRF composition. As these slags were not fully oxidized, and XRF measurements were carried out after fully oxidizing the samples, a correction for the MAC was made using the measured gain in ignition.

Literature survey of EAF slag compositions

An extensive literature survey was conducted to determine the range of EAF slag compositions reported in previous work. These are recorded in Supplementary Table 3. Because of the heterogeneity of the methods used and variations in reporting (sometimes ranges are given) there is some uncertainty about the composition, so they are reported in the main paper as zones, and represented as semi-transparent discs.

Ref	ID in paper	CAO	SIO_2	AL_2O_3	MgO	FeO	Fe_2O_3	Fe.tot	Cr_2O_3	MnO	P_2O_5	Na_2O	K_2O	So_3
**1**	EAF slag 1	45.5	32.2	3.7	5.2	3.3	1.0	4.3	3.3	2.0	0.0	0.0	0.1	
	EAF slag 2	38.8	14.1	6.7	3.9	5.6	20.3	25.9	2.7	5.0	0.2	0.0	0.0	
**2**	EAF Slag	23.9	15.3	7.4	5.1			42.5		4.5				0.1
**3**	EAF Slag	24.2	10.1	5.7	1.9			37.2	2.5	5.1				
**4**	Untreated EAFS	30.8	23.3	6.1	12.0			24.1		1.5	0.6			
**5**	Average	39.6	17.2	4.1	9.2	20.4		24.2		0.7				
**6**	EAFS	53.3	23.0	2.1	7.8	1.1	0.0	1.1			0.1			0.4
**7**	EAF	27.8	16.6	11.0	4.0			33.8	2.2	4.7				
**8**	EAF Type P	29.6	13.0	9.3	3.7			32.8	4.0	5.1				
**9**	EAFS	21.8	18.3	8.3	6.1			37.6	1.8	4.4	0.2	0.4	0.1	0.2
**10**	EAF steel slag	25.6	18.7	2.8	7.5			35.2		0.3		0.3	0.1	
**11**	EAFS1	32.5	17.2	8.0	4.6			30.8		3.8	0.6	0.2		0.3
	EAFS2	25.7	17.9	11.6	3.8			27.5		4.2	0.5	0.1		0.0
**12**	EAF slag	41.7	34.7	6.3	9.1			0.5	3.5	2.1				
**13**	Steel slag	16.9	26.4	4.8	1.9			43.4		2.7		0.2		
**14**	EAFS-1	29.1	6.0	14.1	3.4			27.4	0.7	15.6	1.2	0.1	1.1	
	EAFS-2	24.4	15.4	12.2	2.9			34.4	1.0	5.6	1.2	0.2	1.5	
**15**	EAFs	26.0	14.0	12.0	5.0			35.0	1.3	6.0		0.2	0.1	
**16**	EAFS	32.5	18.1	13.3	2.5	12.0		26.3	1.4	3.9	0.5	0.1	0.0	0.4
**17**	EAFS (0–5 mm)	32.9	20.3	12.2	3.0			22.3	2.0	5.1	0.5			0.4
**18**	EAF	38.9	17.5	4.0	5.0			25.8		2.3	1.5	0.3	0.3	0.5
**19**	steel slag	22.1	5.7	21.9	4.5			24.2	0.7	3.9	0.5	0.4	0.0	0.1
**20**	EAF slag	49.3	28.2	3.9	4.1			4.6	5.5	4.8		0.5	0.0	
**21**	EAF slag	25.9	16.3	8.3	6.9			34.7		5.2	0.3	0.3	0.1	
**22**	Steel slag	40.0	16.0	5.0	5.0			27.0		1.0	2.0			
**23**	EAF slag	30.3	14.6	10.2	3.0			33.3	2.7	4.3	0.0	0.0	0.0	0.0
**24**	EAF steel slag	39.4	17.3	4.2	5.7			25.3					0.3	0.6
**25**	EAF slag	29.2	20.1	8.7	2.8			32.0		4.0		0.0	0.0	
**26**	EAF slag	30.2	19.0	12.7	4.6			25.8	1.6	4.8				
**27**	EAFS	34.9	18.7	11.6	6.7	15.0	16.7	31.7	0.9	2.6	0.3	0.1	0.0	0.7

**28**	S1	26.1	12.4	9.0	3.2			37.5	2.5					
	S2	28.7	10.4	7.3	4.0			39.7	2.0					
**29**	EAFS	27.7	19.1	13.7	2.5			26.8		5.3				
**30**	EAF slag	33.3	19.5	4.9	4.3			25.9			0.4	0.5	0.6	2.3
**31**	EAF slags	22.0	20.0	13.3	7.8			21.3		4.9				
**32**	EAF slag	31.7	17.1	6.1	2.0			36.1		6.3		0.6	0.1	
**33**	EAFS	30.6	16.1	3.8	9.8			31.7				0.6	0.2	0.0
**34**	Steel slag-B	38.0	15.5	4.3	3.5			28.2	0.5	3.6	1.8	0.2	0.1	0.7
**35**	Slag A	20.9	21.1	8.5	14.9			11.4	3.8	2.2		0.1	0.2	
**36**	EAF 0/20 mm	18.3	18.9	5.8	2.5			37.7	0.1	2.6	0.4			
	EAF 14/32 mm	16.5	19.1	5.8	2.0			39.8	0.1	2.8	0.5			
**37**	EAFSS	46.5	12.2	7.2	6.5	23.5	0.9	24.4	0.8	1.3				
**38**	EAFS 1	37.2	28.3	6.4	17.2			3.4	2.5	2.2	0.0	0.1	0.1	0.1
	EAFS 2	26.7	29.1	9.4	18.0			4.4	6.3	2.1	0.0	0.2	0.2	0.1
	EAFS 3	25.8	21.4	5.9	7.8			24.1	6.4	3.3	0.2	0.7	0.9	0.2
	EAFS 4	41.2	15.4	18.9	18.7			2.0	0.6	0.3	0.0	0.0	0.0	0.4
	EAFS-Sb	49.7	36.2	1.3	6.3			2.4	0.6	0.4	0.1	0.1	0.0	1.4
**39**	EAF Slag	34.7	19.1	4.2	6.2			24.2						
**40**	Steel slag	25.6	18.7	2.8	7.5			35.2		0.3		0.3	0.1	
**41**	EVRAZ EAF slag	25.0	8.1	7.6	10.7			44.4	1.5	4.2	0.3	0.0	0.0	
**42**	EAFC slag	32.2	11.4	14.7	3.1			30.5	1.1	4.2			0.1	
**43**	ISS	26.7	20.3	8.9	4.3			32.3	1.7	3.3		0.3	0.2	0.3
**44**	EAFS	27.7	23.9	13.1	3.2			26.4		4.6	0.5	0.0	0.0	
**45**	EAF steel slag	30.7	12.8	12.0	7.7			31.1		0.5	0.4	0.1	0.1	
**46**	EAFS	39.5	8.2	0.7	0.9			38.9		8.1	2.2	0.3	0.3	0.2
**47**	Steel slag	33.0	13.1	5.5	5.0			36.8				0.0	0.0	4.2
**48**	EOS	44.7	33.6	3.8	6.2			0.6	4.9	3.1		0.1		0.5
**49**	EOS	26.1	15.5	11.9	3.4			36.8		6.0				
**50**	EOS slag	40.4	43.0	4.8	2.2			0.6		2.7	3.8			0.2
**51**	EAF 1	25.1	12.2	1.6	7.7			38.5	0.8	5.9	0.6	0.3	0.4	0.6
	EAF 2	30.9	13.4	2.7	2.8			37.9	0.8	4.1	1.4	0.3	0.3	0.7
**52**	63 < d < 90 µm	21.7	18.5	8.5	17.0			12.9	4.0	2.3	0.1	0.2	0.2	0.2
**53**	EAF slag	22.5	16.3	15.1	5.6			22.5	4.6	9.4	0.2			
**54**	EAF oxidizing slag	27.7	15.3	7.4	6.6			31.8						
**55**	EAF Aggregate	34.7	18.1	5.1	5.4			23.2				0.0	0.0	0.0
**56**	EAFS	25.5	16.0	9.2	5.1			25.7				0.2	0.0	0.3
**57**	EAFS	27.7	23.9	13.1	3.2			26.4		4.6	0.5	0.0	0.0	

**58**	EAFS	35.6	18.9	6.9	3.8			31.2	0.5	1.2	0.6	0.0	0.0	0.0
**59**	EAFS	30.3	12.0	7.4	4.9			37.9	1.2	4.5				
**60**	Slag A	20.9	21.1	8.5	14.9			11.4	3.8	2.2		0.1	0.2	
**61**	EAF slag	24.3	12.6	8.8	3.0			40.5		4.7	0.4			
**62**	EAFS	37.9	22.0	7.9	7.5			22.8		2.2				
**63**	EAF slag	29.5	5.7	5.2	4.3			48.5	3.4	5.2	0.3			
**64**	EAFS	26.7	20.9	12.1	3.2			24.5		4.6	0.5	0.0	0.0	
**65**	Ss	55.2	22.4	3.0	8.5			4.4		0.3	0.0	0.1	0.0	
**66**	EAFS	32.9	20.3	12.2	3.0			22.3	2.0	5.0	0.5	0.6		0.4
**67**	EAFS	24.5	12.8	4.9	3.1			46.7	1.2	4.9	0.4	0.2		0.1
**68**	electric arc furnace slag	31.8	17.5	12.3	5.1			22.0	2.7	6.2	0.3		0.0	0.1
**69**	Arc Furnace Slag	38.6	16.1	5.2	14.6			10.3		0.7		0.6	0.1	
**70**	EAFs	38.1	17.1	6.1	6.5			23.6		6.5	0.3	0.0	0.1	
**71**	Electric arc furnace oxidizing slag	26.7	17.7	12.2	5.3			21.2		7.9				
**72**	EAFS S4	22.5	22.1	25.2	11.5			9.9		4.4	0.2	0.0	0.0	0.4
**73**	EAS	24.1	14.2	11.1	3.3			39.9		5.6	0.5			0.0
**74**	Slag	27.9	21.2	5.5	5.7			25.1		0.4	0.5	0.8	0.7	0.3
**75**	modified-EAF slag	30.7	21.6	5.1	10.1			23.9	1.6	4.4				1.3
**76**	EAFS	28.2	17.7	10.2	5.7			28.5	2.4	5.5	0.4	0.2	0.0	0.3
**77**	EAFS	27.0	13.2	13.9	3.8			31.0	2.4	6.4	0.5	0.0	0.1	

Supplementary Table 3 EAF slag compositions reported in the literature.

References cited in Supplementary Table 3:

1. Tossavainen, M. *et al.* Characteristics of steel slag under different cooling conditions. *Waste Manag.* **27**, 1335–1344 (2007).
2. Manso, J. M., Polanco, J. A., Losañez, M. & González, J. J. Durability of concrete made with EAF slag as aggregate. *Cem. Concr. Compos.* **28**, 528–534 (2006).
3. Pellegrino, C. & Gaddo, V. Mechanical and durability characteristics of concrete containing EAF slag as aggregate. *Cem. Concr. Compos.* **31**, 663–671 (2009).
4. Muhmood, L., Vitta, S. & Venkateswaran, D. Cementitious and pozzolanic behavior of electric arc furnace steel slags. *Cem. Concr. Res.* **39**, 102–109 (2009).
5. Abu-Eishah, S. I., El-Dieb, A. S. & Bedir, M. S. Performance of concrete mixtures made with electric arc furnace (EAF) steel slag aggregate produced in the Arabian Gulf region. *Constr. Build. Mater.* **34**, 249–256 (2012).

6. Li, J., Yu, Q., Wei, J. & Zhang, T. Structural characteristics and hydration kinetics of modified steel slag. *Cem. Concr. Res.* **41**, 324–329 (2011).

7. Pellegrino, C., Cavagnis, P., Faleschini, F. & Brunelli, K. Properties of concretes with Black/Oxidizing Electric Arc Furnace slag aggregate. *Cem. Concr. Compos.* **37**, 232–240 (2013).

8. Pasetto, M. & Baldo, N. Experimental evaluation of high performance base course and road base asphalt concrete with electric arc furnace steel slags. *J. Hazard. Mater.* **181**, 938–948 (2010).

9. Etxeberria, M., Pacheco, C., Meneses, J. M. & Berridi, I. Properties of concrete using metallurgical industrial by-products as aggregates. *Constr. Build. Mater.* **24**, 1594–1600 (2010).

10. Ameri, M., Hesami, S. & Goli, H. Laboratory evaluation of warm mix asphalt mixtures containing electric arc furnace (EAF) steel slag. *Constr. Build. Mater.* **49**, 611–617 (2013).

11. Arribas, I., Santamaría, A., Ruiz, E., Ortega-López, V. & Manso, J. M. Electric arc furnace slag and its use in hydraulic concrete. *Constr. Build. Mater.* **90**, 68–79 (2015).

12. Adegoloye, G., Beaucour, A.-L., Ortola, S. & Noumowé, A. Concretes made of EAF slag and AOD slag aggregates from stainless steel process: Mechanical properties and durability. *Constr. Build. Mater.* **76**, 313–321 (2015).

13. Roslan, N. H., Ismail, M., Abdul-Majid, Z., Ghoreishiamiri, S. & Muhammad, B. Performance of steel slag and steel sludge in concrete. *Constr. Build. Mater.* **104**, 16–24 (2016).

14. Luxán, M. P., Sotolongo, R., Dorrego, F. & Herrero, E. Characteristics of the slags produced in the fusion of scrap steel by electric arc furnace. *Cem. Concr. Res.* **30**, 517–519 (2000).

15.. Monosi, S., Ruello, M. L. & Sani, D. Electric arc furnace slag as natural aggregate replacement in concrete production. *Cem. Concr. Compos.* **66**, 66–72 (2016).

16. Iacobescu, R. I., Koumpouri, D., Pontikes, Y., Saban, R. & Angelopoulos, G. N. Valorisation of electric arc furnace steel slag as raw material for low energy belite cements. *J. Hazard. Mater.* **196**, 287–294 (2011).

17. Santamaría, A. *et al.* Self-compacting concrete incorporating electric arc-furnace steelmaking slag as aggregate. *Mater. Des.* **115**, 179–193 (2017).

18. Kavussi, A. & Qazizadeh, M. J. Fatigue characterization of asphalt mixes containing electric arc furnace (EAF) steel slag subjected to long term aging. *Constr. Build. Mater.* **72**, 158–166 (2014).

19. Fällman, A.-M. Leaching of chromium and barium from steel slag in laboratory and field tests — a solubility controlled process? *Waste Manag.* **20**, 149–154 (2000).

20. Baciocchi, R., Costa, G., Polettini, A. & Pomi, R. Effects of thin-film accelerated carbonation on steel slag leaching. *J. Hazard. Mater.* **286**, 369–378 (2015).

21. Lam, M. N.-T., Le, D.-H. & Jaritngam, S. Compressive strength and durability properties of roller-compacted concrete pavement containing electric arc furnace slag aggregate and fly ash. *Constr. Build. Mater.* **191**, 912–922 (2018).

22. Fakhri, M. & Ahmadi, A. Recycling of RAP and steel slag aggregates into the warm mix asphalt: A performance evaluation. *Constr. Build. Mater.* **147**, 630–638 (2017).

23. Pomaro, B. *et al.* Gamma-ray shielding properties of heavyweight concrete with Electric Arc Furnace slag as aggregate: An experimental and numerical study. *Constr. Build. Mater.* **200**, 188–197 (2019).

24. Masoudi, S., Abtahi, S. M. & Goli, A. Evaluation of electric arc furnace steel slag coarse aggregate in warm mix asphalt subjected to long-term aging. *Constr. Build. Mater.* **135**, 260–266 (2017).

25. Teo, P.-T., Anasyida, A. S., Basu, P. & Nurulakmal, M. S. Recycling of Malaysia's electric arc furnace (EAF) slag waste into heavy-duty green ceramic tile. *Waste Manag.* **34**, 2697–2708 (2014).

26. González-Ortega, M. A. *et al.* Radiological protection and mechanical properties of concretes with EAF steel slags. *Constr. Build. Mater.* **51**, 432–438 (2014).

27. Ozturk, M. *et al.* Experimental work on mechanical, electromagnetic and microwave shielding effectiveness properties of mortar containing electric arc furnace slag. *Constr. Build. Mater.* **165**, 58–63 (2018).

28. Mombelli, D. *et al.* The efficiency of quartz addition on electric arc furnace (EAF) carbon steel slag stability. *J. Hazard. Mater.* **279**, 586–596 (2014).

29. Fuente-Alonso, J. A., Ortega-López, V., Skaf, M., Aragón, Á. & San-José, J. T. Performance of fiber-reinforced EAF slag concrete for use in pavements. *Constr. Build. Mater.* **149**, 629–638 (2017).

30. Rooholamini, H., Sedghi, R., Ghobadipour, B. & Adresi, M. Effect of electric arc furnace steel slag on the mechanical and fracture properties of roller-compacted concrete. *Constr. Build. Mater.* **211**, 88–98 (2019).

31. Kim, H.-S. *et al.* Valorization of electric arc furnace primary steelmaking slags for cement applications. *Waste Manag.* **41**, 85–93 (2015).

32. Papachristoforou, M., Anastasiou, E. K. & Papayianni, I. Durability of steel fiber reinforced concrete with coarse steel slag aggregates including performance at elevated temperatures. *Constr. Build. Mater.* **262**, 120569 (2020).

33. Khan, K. & Amin, M. N. Influence of fineness of volcanic ash and its blends with quarry dust and slag on compressive strength of mortar under different curing temperatures. *Constr. Build. Mater.* **154**, 514–528 (2017).

34. Zhang, N., Wu, L., Liu, X. & Zhang, Y. Structural characteristics and cementitious behavior of basic oxygen furnace slag mud and electric arc furnace slag. *Constr. Build. Mater.* **219**, 11–18 (2019).

35. Češnovar, M., Traven, K., Horvat, B. & Ducman, V. The potential of ladle slag and electric arc furnace slag use in Synthesizing alkali activated materials; the influence of curing on mechanical properties. *Materials (Basel).* **12**, 1173 (2019).

36. Autelitano, F. & Giuliani, F. Electric arc furnace slags in cement-treated materials for road construction: Mechanical and durability properties. *Constr. Build. Mater.* **113**, 280–289 (2016).

37. Nikolić, I. *et al.* Kinetics of electric arc furnace slag leaching in alkaline solutions. *Constr. Build. Mater.* **108**, 1–9 (2016).

38. Andreas, L., Diener, S. & Lagerkvist, A. Steel slags in a landfill top cover – Experiences from a full-scale experiment. *Waste Manag.* **34**, 692–701 (2014).

39. Manjunath, R., Narasimhan, M. C., Umesh, K. M., Shivam Kumar & Bala Bharathi, U. K. Studies on development of high performance, self-compacting alkali activated slag concrete mixes using industrial wastes. *Constr. Build. Mater.* **198**, 133–147 (2019).

40. Goli, H., Hesami, S. & Ameri, M. Laboratory Evaluation of Damage Behavior of Warm Mix Asphalt Containing Steel Slag Aggregates. *J. Mater. Civ. Eng.* **29**, 04017009 (2017).

41. Okochi, N. C. & McMartin, D. W. Laboratory investigations of stormwater remediation via slag: Effects of metals on phosphorus removal. *J. Hazard. Mater.* **187**, 250–257 (2011).

42. Liapis, I. & Papayianni, I. Advances in chemical and physical properties of electric arc furnace carbon steel slag by hot stage processing and mineral mixing. *J. Hazard. Mater.* **283**, 89–97 (2015).

43. Cristelo, N., Coelho, J., Miranda, T., Palomo, Á. & Fernández-Jiménez, A. Alkali activated composites – An innovative concept using iron and steel slag as both precursor and aggregate. *Cem. Concr. Compos.* **103**, 11–21 (2019).

44. Santamaría-Vicario, I., Rodríguez, A., Gutiérrez-González, S. & Calderón, V. Design of masonry mortars fabricated concurrently with different steel slag aggregates. *Constr. Build. Mater.* **95**, 197–206 (2015).

45. Pattanaik, M. L., Choudhary, R. & Kumar, B. Clogging evaluation of open graded friction course mixes with EAF steel slag and modified binders. *Constr. Build. Mater.* **159**, 220–233 (2018).

46. Khater, H. M. Hybrid slag geopolymer composites with durable characteristics activated by cement kiln dust. *Constr. Build. Mater.* **228**, 116708 (2019).

47. Heniegal, A. M., Amin, M. & Youssef, H. Effect of silica fume and steel slag coarse aggregate on the corrosion resistance of steel bars. *Constr. Build. Mater.* **155**, 846–851 (2017).

48. Kuo, W.-T. & Shu, C.-Y. Effect of particle size and curing temperature on expansion reaction in electric arc furnace oxidizing slag aggregate concrete. *Constr. Build. Mater.* **94**, 488–493 (2015).

49. Lee, H.-S., Lim, H.-S. & Ismail, M. A. Quantitative evaluation of free CaO in electric furnace slag using the ethylene glycol method. *Constr. Build. Mater.* **131**, 676–681 (2017).

50. Kuo, W.-T., Shu, C.-Y. & Han, Y.-W. Electric arc furnace oxidizing slag mortar with volume stability for rapid detection. *Constr. Build. Mater.* **53**, 635–641 (2014).

51. Cárdenas Balaguera, C. A. & Gómez Botero, M. A. Characterization of steel slag for the production of chemically bonded phosphate ceramics (CBPC). *Constr. Build. Mater.* **241**, 118138 (2020).

52. Traven, K., Češnovar, M. & Ducman, V. Particle size manipulation as an influential parameter in the development of mechanical properties in electric arc furnace slag-based AAM. *Ceram. Int.* **45**, 22632–22641 (2019).

53. Roy, S., Miura, T., Nakamura, H. & Yamamoto, Y. Investigation on applicability of spherical shaped EAF slag fine aggregate in pavement concrete – Fundamental and durability properties. *Constr. Build. Mater.* **192**, 555–568 (2018).

54. Koh, T., Shin, M., Bae, Y. & Hwang, S. Structural performances of an eco-friendly prestressed concrete sleeper. *Constr. Build. Mater.* **102**, 445–454 (2016).

55. Jagadisha, A., Rao, K. B., Nayak, G. & Kamath, M. Influence of nano-silica on the microstructural and mechanical properties of high-performance concrete of containing EAF aggregate and processed quarry dust. *Constr. Build. Mater.* **304**, 124392 (2021).

56. Hafez, H., Kassim, D., Kurda, R., Silva, R. V. & de Brito, J. Assessing the sustainability potential of alkali-activated concrete from electric arc furnace slag using the ECO2 framework. *Constr. Build. Mater.* **281**, 122559 (2021).

57. García-Cuadrado, J., Rodríguez, A., Cuesta, I. I., Calderón, V. & Gutiérrez-González, S. Study and analysis by means of surface response to fracture behavior in lime-cement mortars fabricated with steelmaking slags. *Constr. Build. Mater.* **138**, 204–213 (2017).

58. Rashad, A. M., Khafaga, S. A. & Gharieb, M. Valorization of fly ash as an additive for electric arc furnace slag geopolymer cement. *Constr. Build. Mater.* **294**, 123570 (2021).

59. Sosa, I. *et al.* Durability of high-performance self-compacted concrete using electric arc furnace slag aggregate and cupola slag powder. *Cem. Concr. Compos.* **127**, 104399 (2022).

60. Lancellotti, I. *et al.* Alkali Activation of Metallurgical Slags: Reactivity, Chemical Behavior, and Environmental Assessment. *Materials (Basel).* **14**, 639 (2021).

61. Gallego, J., Gulisano, F., Contreras, V. & Páez, A. The crucial effect of re-compaction energy on the healing response of hot asphalt mortars heated by microwaves. *Constr. Build. Mater.* **285**, 122861 (2021).

62. Shiha, M., El-Badawy, S. & Gabr, A. Modeling and performance evaluation of asphalt mixtures and aggregate bases containing steel slag. *Constr. Build. Mater.* **248**, 118710 (2020).

63. Menad, N.-E., Kana, N., Seron, A. & Kanari, N. New EAF Slag Characterization Methodology for Strategic Metal Recovery. *Materials (Basel).* **14**, 1513 (2021).

64. García-Cuadrado, J., Santamaría-Vicario, I., Rodríguez, A., Calderón, V. & Gutiérrez-González, S. Lime-cement mortars designed with steelmaking slags as aggregates and validation study of their properties using mathematical models. *Constr. Build. Mater.* **188**, 210–220 (2018).

65. Rahou, J., Rezqi, H., El Ouahabi, M. & Fagel, N. Characterization of Moroccan steel slag waste: The potential green resource for ceramic production. *Constr. Build. Mater.* **314**, 125663 (2022).

66. Ortega-López, V., Revilla-Cuesta, V., Santamaría, A., Orbe, A. & Skaf, M. Microstructure and Dimensional Stability of Slag-Based High-Workability Concrete with Steelmaking Slag Aggregate and Fibers. *J. Mater. Civ. Eng.* **34**, (2022).

67. Yue, H. *et al.* Investigation on applicability of spherical electric arc furnace slag as fine aggregate in superplasticizer-free 3D printed concrete. *Constr. Build. Mater.* **319**, 126104 (2022).

68. Terrones-Saeta, J. M., Suárez-Macías, J., Moreno-López, E. R. & Corpas-Iglesias, F. A. Determination of the Chemical, Physical and Mechanical Characteristics of Electric Arc Furnace Slags and Environmental Evaluation of the Process for Their Utilization as an Aggregate in Bituminous Mixtures. *Materials (Basel).* **14**, 782 (2021).

69. Khater, G. A., Nabawy, B. S., El-Kheshen, A. A., Abdel Latif, M. A.-B. & Farag, M. M. Use of Arc Furnace Slag and Ceramic Sludge for the Production of Lightweight and Highly Porous Ceramic Materials. *Materials (Basel).* **15**, 1112 (2022).

70. Hernandez-Fernandez, N., García-Mejía, T. A., Ramírez-Zamora, R. M., Ochoa-Diaz, R. & Ossa-Lopez, A. Effect of application of metallurgical slag powders on physical and rheological properties of asphalt. *Constr. Build. Mater.* **292**, 123432 (2021).

71. Lee, Y.-J. *et al.* Bond performance of reinforced concrete beams with electric arc furnace slag aggregates. *Constr. Build. Mater.* **244**, 118366 (2020).

72. Bullerjahn, F. & Bolte, G. Composition of the reactivity of engineered slags from bauxite residue and steel slag smelting and use as SCM for Portland cement. *Constr. Build. Mater.* **321**, 126331 (2022).

73. Yuan, T.-F., Hong, S.-H., Choi, J.-S. & Yoon, Y.-S. Evaluation on the Microstructure and Durability of High-Strength Concrete Containing Electric Arc Furnace Oxidizing Slag. *Materials (Basel).* **14**, 1304 (2021).

74. Ziaee, S. A., Moghadas Nejad, F., Dareyni, M. & Fakhri, M. Evaluation of rheological and mechanical properties of hot and warm mix asphalt mixtures containing Electric Arc Furnace Slag using gyratory compactor. *Constr. Build. Mater.* **378**, 131042 (2023).

75. Sukmak, P. *et al.* The potential of industrial waste: Electric arc furnace slag (EAF) as recycled road construction materials. *Constr. Build. Mater.* **368**, 130393 (2023).

76. Lamaa, G., Suescum-Morales, D., Duarte, A. P. C., Silva, R. V. & de Brito, J. Optimising the Performance of CO2-Cured Alkali-Activated Aluminosilicate Industrial By-Products as Precursors. *Materials (Basel).* **16**, 1923 (2023).

77. Benavides, D., Barra Bizinotto, M., López, T. & Aponte, D. Study of adhesion between steel slag aggregates and bitumen taking into consideration internal factors influencing moisture damage. *Constr. Build. Mater.* **367**, 130369 (2023).

Experimental notes

Leaching

Early experiments in this study were conducted with an aluminium oxide lining in a 250 kg furnace. As reported in the main paper, this lining proved unsuitable for re-clinkering over molten steel and led to uncontrolled leaching. To illustrate the effect, Supplementary Figure 1 shows the difference in oxide composition between the input flux and the output slag after fluxing over molten steel in three different crucibles. The effect of leaching from the Alumina crucible can be seen from the large increase in Al_2O_3 in the samples prepared with it. Unlike the results reported in the main paper using MgO or graphite crucibles, the aluminium content of the slag is much larger than that of the flux because of leaching.

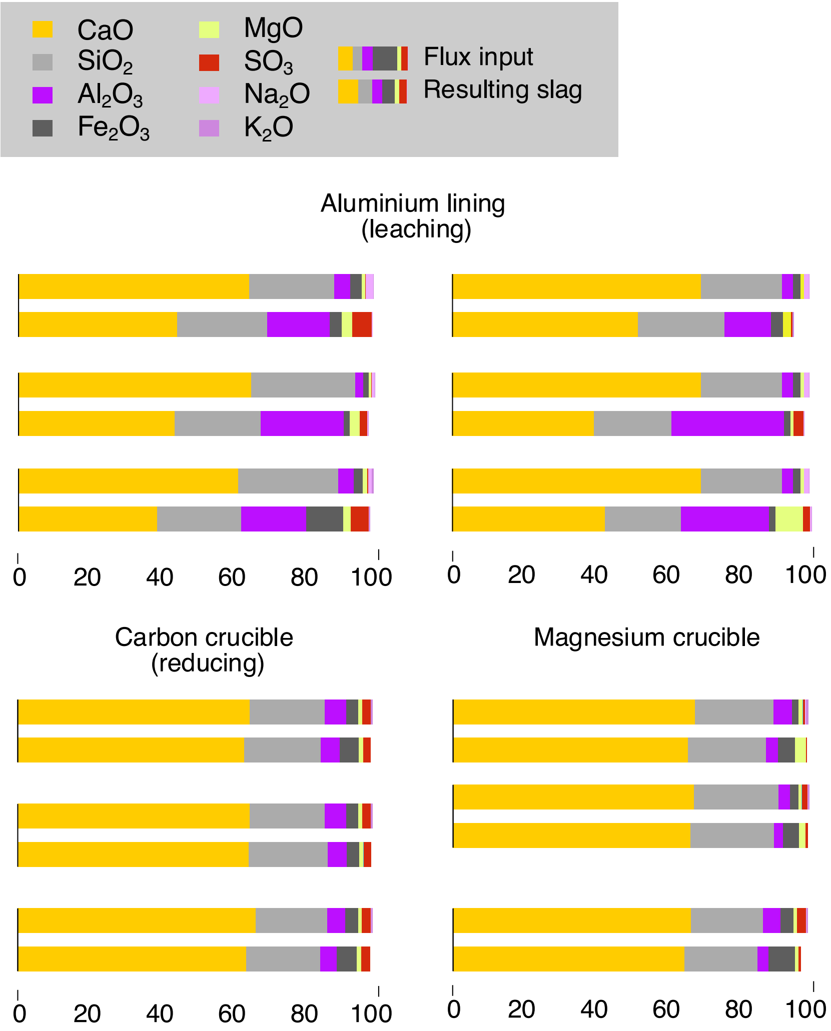

Supplementary Figure 1: Oxide composition difference between flux and slag when using three different crucibles. The figure shows the outcomes of six trials in a crucible with aluminium lining, three with a carbon crucible and three with a magnesium crucible. Each trial is represented with a pair of results, of which the upper bar shows the oxide

composition of the input flux and the lower bar shows the oxide composition of the resulting slag. The effect of leaching in the aluminium crucible is demonstrated by the significant changes in composition between flux and slag in the six pairs of results in the upper part of the figure.

Sulphur in steel and slag

The major difference between cement paste and the lime flux added in EAF operations, is the inclusion of SiO_2, Al_2O_3, Fe_2O_3 and SO_3. SiO_2, Al_2O_3 and iron oxides are compatible with the slag and are usually present in scrap and slag due to oxidation reactions. Further, Si and Al cannot easily be reduced in EAF operating conditions used to melt steel. Thus Si and Al remain as oxides in the slag and will not affect steel. The effect of having SO_3 in flux is unknown to steelmaking, but it is volatile at the operating temperatures of EAFs.

High sulphur content has a negative impact on the mechanical properties of steel, so it is typically limited to less than 0.05%. To test the risk of this with the new process, in some of our experiments, we used a flux to steel ratio at least 7 times higher than a typical flux addition in the EAF process, to increase the potential transfer of sulphur into the system. Nevertheless, the resulting sulphur content of the steel was no more than 0.08% in all cases, so with more conventional flux:steel ratios, we do not anticipate a problem.

In detail, in trial MO2-1, around 150g of flux was added to 500g of steel. The SO_3 content in the flux was 2.4%, resulting in 1.24g of sulphur in the flux. Of that, 0.31g stayed in the slag, 0.35g transferred to the steel and the remaining 0.58g was volatile. The resulting sulphur content of steel was therefore around 0.07%.

In the combined double-slagging trials MO2-6, (i.e. MO2-6a and MO2-6b), around 300g of flux was added in total, amounting to 2.4g of sulphur added, of which 0.4g went to steel, 1.1g to slag and 0.9g was volatile, leading to a sulphur content in the steel of 0.08%.

In reducing conditions with a graphite crucible, the SO_3 content remained almost unchanged with flux and slag, indicating almost all the S stayed with the slag and hardly any sulphur went to steel.

Nevertheless, the effect of sulphate content in the flux on the partition of sulphur in EAF must be studied further, and the costs of the additional sulphur load must be investigated.

Performance

All the strength tests were performed according to EN 196-1 and can be found in Supplementary Table 4.

	Compressive strength (MPa)		
	**2 day**	**7 day**	**28 day**
Commercial cement	29.8	51.8	62.2
	31.6	51.3	66.5
	31.7	-	66.7
Commercial cement LC3	19.98	47.30	67.72
	19.84	48.29	68.29
	19.47	47.74	67.97
Commercial clinker	21.51	35.41	57.58
	23.82	37.21	52.74
	21.44	38.46	53.07
Commercial clinker LC3	15.53	44.36	57.06
	15.11	44.13	58.21
	15.67	44.17	56.79
High Alite (GC80-1a)	23.10	36.65	47.74
	24.54	35.54	47.27
	24.69	40.22	48.23
High Alite LC3	18.27	46.62	61.58
	17.46	45.93	59.05
	17.94	43.15	61.40
Medium Alite (GC80-1b)	17.49	27.04	40.46
	17.40	27.21	41.92
	17.67	27.59	39.65
Medium Alite LC3	12.75	37.43	51.73
	12.53	37.19	51.75
	12.53	39.34	49.77

Supplementary Table 4: Strength of the samples at 2, 7 and 28 days, tested according to EN 196-1.

Under-sulphation in strength tests

The cements prepared for the strength tests were under-sulphated. As our production volumes are currently constrained by equipment and labour availability, figure 2A in the main paper presents updated calorimetry data (used to anticipate the setting time) for properly sulphated samples, but we have not had the material to repeat the strength tests shown in figure 2C. For completeness, we therefore present here the calorimetric curves, both instant and cumulative, for the samples used to create figure 2C. The earlier results show a large early peak due to aluminate hydration overlayed over the main (silicate) peak.

When, the cement is properly sulphated, as with the samples in figure 2A, this peak occurs after the silicate peak, and is smaller.

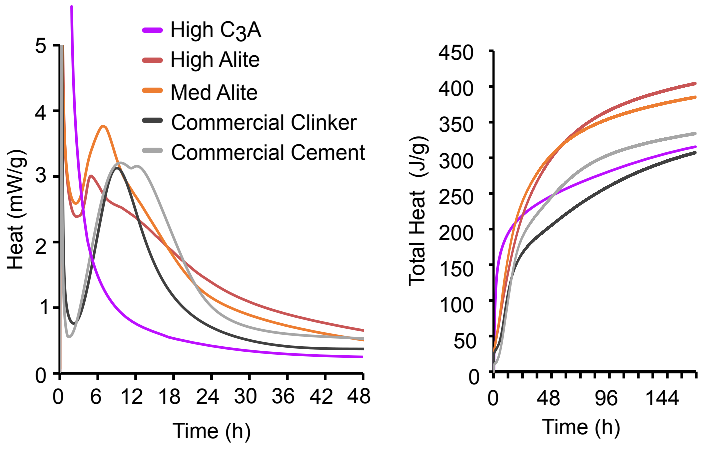

Supplementary Figure 2 Calorimetry results for the under-sulfated samples used to create the strength tests of figure 2C in the main paper.

Emissions and economic analysis of electric cement recycling in the UK

Boundaries and assumptions

To anticipate the cost and emissions impact of making Portland cement by electric-recycling of cement paste, we assume that production occurs in a ~500kt/yr Electric Arc Furnace, creating 150kt/yr of slag, all of which is converted to clinker. We assume that the masses of flux and slag are the same, although generally, the mass of slag will be higher, as it combines the flux with impurities in the steel scrap.

The analysis aims to capture all changes to existing processing. Concrete and Demolition Waste is already collected, transported, processed and sold, so only the additional costs of improved separation are included, and are offset by higher revenues from purer recycled aggregates and a new stream of revenue from sand recycling.

The emissions figures are estimated from global average data, to avoid confusion from regional variations in electricity generation, or transport configuration. However, the cost figures are estimated for the UK in 2023, to ensure consistent comparison based on local energy, labour, cement and raw material prices.

The cost of capital for all additional equipment purchased to enable electric cement recycling is taken as 25%.

Given that cement production is largely localised, while steel recycling entails some trade of scrap, it has been assumed that recycled cement paste (RCP) must be transported 320 km to the electric arc furnace, and 320km from there to a cement distribution centre, with 80% of the transport occurring by ship and 20% by road. These distances would be typical for the UK, which currently has 11 cement plants and 2 large EAFs.

The key proportions, emissions factors and cost basis of the analysis are presented in Supplementary Table 5:

**Inputs**	**Emissions intensity (global)**	**Units**	**Cost (uk)**	**Units**	**Note**
Calcined clay	0.25	tonnes CO2/tonne clay	65	£/tonne	1
Anhydrous Calcium Sulphate Powder	0.06	tonnes CO2/tonne gypsum	200	£/tonne	2
Lime Flux	1.1	tonnes CO2/tonne lime	£24	per tonne	3
Sand	0.005	tonnes CO2/tonne sand	£35	per tonne	4
Ground limestone	0.03	tonnes CO2/tonne limestone	£24	Per tonne	5
Binder for pelletising	1.1	tonnes CO2/tonne binder	£12	per tonne	6
Electricity generation	0.00013	tonnes CO2/MJ	0.02	£/MJ	7
Road transport (UK)	0.000125	tonnes CO2/tonne km	£0.08	£/tonne-km	8
Ship transport (UK)	0.000005	tonnes CO2/tonne km	£0.0006	£/tonne-km	9
Additional Operators (UK)			£80,000	Per person year	10

Supplementary Table 5: Proportions, emissions factors and cost basis of the analysis.

Notes to Supplementary Table 5:

1. The cost and emissions of calcined clay are expected to be around half the cost and emissions of OPC as production requires half the temperature for calcining. However, BGS fact sheets for Kaolin (2009) and brick-clay (2022) suggest the price might be nearer to £100 per tonne, and Alibaba spot prices are around $100-200 per tonne, so the £65/tonne estimate used here may be optimistic. Diaz et al (2017) table 1 give an emissions factor of 0.2 tonnes CO_2/tonne clay for flash calcining. Berriel et al. (2016) estimate industrial energy requirements of 23 kg of crude oil and 16kWh of electricity for calcining 300kg of clay while producing one tonne of LC3-50 cement. Using global average emissions factors this leads to the estimate of 0.25 tonnes CO_2/tonne clay used in the table. As clay calcination has no process emissions, this emissions factor could be reduced to zero if calcination were

powered by emissions-free electricity. Our calculation below for scenario 2b, includes this assumption, leading to the maximum possible abatement from use of LC^3-CEC.

2. The emissions factor is taken from various secondary papers citing a 2008 WRAP study by Fisher; the cost estimate is uncertain and based on spot prices on alibaba.com, albeit Hanein et al (2018) estimate £10/tonne for gypsum; Riveiro et al (2016) in Table S6 estimate 474 kt CO_2 to mine and process the gypsum required for 10.6 Mt content in plasterboard - giving an emissions factor of 0.05 tonnes CO_2/tonne gypsum

3. Meier et al (2005) quote a 1998 source to estimate 0.95-1.20 t CO_2/tonne of lime. We used 1.1 as the middle of this range.

4. Emissions data from Akan et al (2017) citing several sources.

5. Emissions from Proske et al (2016), sourced in turn from the GaBi database; price estimated at half the price of lime flux due to absence of processing

6. Assumed to be half the price of lime flux, with the same emissions - no data found.

7. Current world average is 0.460 tonnes CO_2/MWh (IEA World Energy Outlook (2022) page 45); Price estimate based on current UK Industrial prices. (£0.08/kWh, 1kWh=3.6MJ, https://energy-stats.uk/wholesale-energy-pricing/)

8. From https://www.roadhaulageservices.com/haulage-rates/: £2.50 per mile for a 20 tonne load (1.6km=1 mile); IPCC AR5 table 8.5 reports emissions of ~125g/tonne km for large freight;

9. Bulk carrier rates (FT, 2021): £40,000/day for 180,000 tonnes, average speed 15mph, assume 16 hours moving and 8 hours loading/unloading gives 240 miles per day, hence £0.001 per tonne mile (1.6km=1 mile). IPCC AR5 table 8.5 reports ~5g/tonne km for large freight;

10. Estimated

Detailed Calculation

Estimates are made of the cost and emissions consequences of four recipes for recycled Cambridge Electric Cement (CEC): (a) a “pure” blend with 95% CEC clinker and 5% gypsum and (b) an LC3-50 blend with 50% clinker, 30% calcined clay, 15% ground limestone and 5% gypsum, powered by (1) a grid having today’s global average electricity emissions and (2) assuming complete decarbonisation of the electricity grid.

The detailed calculation for recipe 1b (LC3-50 CEC with today’s grid) is provided in Supplementary Table 6. The equivalent calculations for the other recipes follow directly from application of the assumptions in Supplementary Table 5.

	Additional Resource	Units /tonne clinker	Electricity required (MJ/tonne)	Emissions increase (tonnes CO_2)	Cost increase (£)	Note
Changed Aggregate separation						
Heating by 450K prior to crushing and sieving	2450	MJ	2450	0.31	£64.89	1
Additional revenue from improved aggregate					£0	2
Additional revenue from sand recycling					-£35.00	3
Avoided disposal charge for fines					-£3.50	4
Additional margin for Separator					£10.00	5
**One tonne RCP supplied to flux-blending**				**0.31**	**£36.39**	
Flux Blending						
55% RCP	0.55	tonnes		0.17	£20.01	
25% conventional lime flux	0.25	tonnes		0.28	£6.00	6
10% sand	0.1	tonnes		0.001	£3.50	7
10% Binder for pelletising flux	0.1	tonnes		0.11	£1.20	
Pelletising, conditioning and validation			2.5	0.00	£8.09	8
**One tonne flux ready for EAF operation**				**0.56**	**£38.80**	
EAF operation						
Savings from avoiding conventional lime flux	-1	tonnes		-1.1	-£24	9
Installation/operation of slag line			160	0.020	£10.76	10
Slag-grinding			285	0.036	£8.53	11
**One tonne of new clinker ready for blending**				**-0.49**	**£34.09**	
Cement blending and distribution						
50% ground CEC clinker	0.5	tonnes		-0.24	£17.05	
30% calcined clay	0.3	tonnes		0.08	£19.50	
15% ground limestone	0.15	tonnes		0.005	£1.80	
5% anhydrous Calcium Sulphate powder	0.05	tonnes		0.003	£10.00	
Transport of flux to EAF	320	km		0.01	£5.16	
Transport of cement to distribution centre	320	km		0.01	£5.16	
**One tonne of Cambridge Electric Cement**				**-0.14**	**£58.67**	12

Supplementary Table 6: Detailed calculation of emissions and costs for recipe 1b for LC3-50 electric recycled cement made with today's global grid.

Notes to Supplementary Table 6:

1. Following the analysis of Prajapati et al (2021), the treatment here assumes that prior to crushing and sieving, the CDW is heated, to enable high quality separation and a new revenue stream for recycled sand. The specific heat capacity of concrete is ~880 J/kgK (so 0.88MJ/tonne/K). We assume a 450K temperature increase (with electrical power) with efficiency of 85% gives ~466MJ per tonne heated. Assuming that concrete feedstock has mass ratios of 4:2:1 for aggregate:sand:cement, and that the resulting RCP has a 75% purity (concentration of cement) then 5.25 tonnes of CDW must be processed to deliver 1 tonne of recycled cement paste (RCP) (Figure 3B). The capital cost of the additional furnace and crushing/separating improvements is estimated at £6m. One additional operator is required.

2. There could be a price premium for clean recycled aggregate compared with recycled aggregate today of around £10 per tonne, but this would require further processing of the crushed aggregate. It is therefore excluded from this analysis.

3. New revenue stream for separator estimated as 50% of the price for new sales of sand at £15/tonne (half the price of new sand). This value is uncertain, so Supplementary Table 6 demonstrates a sensitivity analysis, varying the price of recycled sand between 0% and 100% of the price of new sand.

4. Based on UK Industrial landfill charges

5. A margin of approximately 25% of costs is added here to ensure a strong business case for commercial production of RCP.

6. This level of substitution follows the results presented in the paper, although there is likely to be scope for optimisation in future.

7. The results show that this may be necessary, although in reality it would be delivered through a reduced quality separation of RCP

8. Costs assume capital purchase of £3m for the press, energy for operation, and one additional operator. Energy estimated as half the energy of crushing taken from Czigler et al (2020)

9. Assumes that this saving is attributed to CEC where it would more likely be shared with the EAF steel

10. Assumes £4m cost for the slag line plus one operator plus energy

11. Assumes £1m cost for new grinding equipment plus one operator plus energy

12. This is the estimate of cost, without any profit margin – which depends on the comparison with existing cement, and the opportunity to attract a price premium arising from market preferences for lower emissions cement.

The results of the emissions calculation are compared with emissions and costs for ordinary Portland cement, and LC3-50 cement. Portland Cement is assumed to cause 0.80 t CO_2/tonne cement and cost £100/tonne (Global Cement, 2011). LC3-50 is assumed to combine 50% OPC with 30% calcined clay and 15% ground limestone and 5% gypsum (Bishnoi et al. 2014, Scrivener et al., 2018) which using the data in Supplementary Table 5, leads to emissions of 0.48 t CO_2/tonne cement. UK consumers are currently prepared to pay a price premium for lower carbon cement which, based on anecdotal evidence in the industry, we estimate to give a price of around £120/tonne for the 40% reduction in emissions in LC3-50. Potentially, this price premium will be higher for the greater emissions savings of electrically recycled cement and may rise higher as government policies impose stricter obligations on emissions (whether through a carbon tax or other mechanism) giving a greater market attraction to recycled cement.

Results

The four recipes for electrically recycled cement are compared to today's OPC and LC3-50 cement in Supplementary Table 7.

			**Today's grid**		**Zero-emissions grid**	
Emissions per tonne of cement (not clinker)	*OPC*	*LC3-50*	*1a "pure" blend*	*1b LC3-50 blend*	*2a "pure" blend*	*2b LC3-50 blend*
Separating RCP			0.16	0.09	0.00	0.00
Blending and pelletising flux			0.37	0.19	0.37	0.19
Producing clinker at EAF slag line			-0.99	-0.52	-1.05	-0.55
Blending clinker to cement			0.00	0.08	0.00	0.01
Total transport			0.02	0.02	0.02	0.02
**Total emissions**	**0.8**	**0.54**	**-0.44**	**-0.14**	**-0.66**	**-0.33**
Cost per tonne of cement (not clinker)						
Separating RCP			19.01	10.01	19.01	10.01
Blending and pelletising flux			17.85	9.39	17.85	9.39
Producing clinker at EAF slag line			-4.48	-2.36	-4.48	-2.36
Blending clinker to cement			10.00	31.30	10.00	31.30
Total transport			10.32	10.32	10.32	10.32
**Total cost**	**£100**	**£120**	**£52.71**	**£58.67**	**£52.71**	**£58.67**

Supplementary Table 7: Comparison of electrically recycled cement with current alternatives.

(The negative costs and emissions for producing clinker at the slag line in Supplementary Table 7 arise from the saving from avoiding use of conventional lime flux in the EAF.) Following note 9 above, the emissions calculation assumes that all benefits of substituting RCP for lime flux are credited to the production of cement. Hence, the emissions for

“producing clinker at EAF slag line” above are all negative. In reality, some negotiation would take place as to how this benefit should be attributed between steel and cement production.

Figure 3A in the main paper is drawn from the numbers in Supplementary Table 5. N.b. the costs for the three recipes based on the new electric cement do not include a profit margin – where those for OPC and LC3-50 do.

Supplementary Table 8 shows the sensitivity of the total costs and emissions from Supplementary Table 7 to the estimated price of recycled sand (note 3 to Supplementary Table 6). The results indicate that this price makes a significant difference to the profit potential of the new recycled cement, but even without any revenue, the new cement is competitive with existing OPC.

	**REFERENCE PRICES**		**TODAY’S GRID**		**ZERO-EMISSIONS GRID**	
Cost sensitivity to recycled sand value	OPC	"Green" OPC	1a “pure” blend	1b LC3-50 blend	2a “pure” blend	2b LC3-50 blend
Recycled sand worth 0% new sand	£100.00	£120.00	£70.99	£68.29	£70.99	£68.29
Recycled sand worth 50% new sand	£100.00	£120.00	£52.71	£58.67	£52.71	£58.67
Recycled sand worth 100% new sand	£100.00	£120.00	£34.42	£49.04	£34.42	£49.04

Supplementary Table 8: Sensitivity of CEC cost to the value of recycled sand.

Discussion

The energy for making Cambridge Electric Cement (CEC) (over and above that required for electric arc steel making) is dominated by heating prior to crushing for advanced RCP separation. We have assumed the worst case here but anticipate that with development the total energy input will be reduced.

The emissions of pure CEC with today's grid are dominated by electricity generation to power the heating required prior to separation. As the grid is decarbonised these will drop towards zero. The question of how the emissions benefit of using RCP instead of lime-flux are shared between the cement and steel industry is political and will evolve over time.

Once the grid is decarbonised, the emissions of making CEC clinker are almost entirely determined by the emissions associated with any conventional lime-flux blended into the RCP. The results of the paper suggest that this may be reduced to around 5% - although for now the results above assume 25%.

If CEC clinker is blended with calcined clay, while 25% lime is blended with RCP, the emissions of the resulting cement are increased compared with "pure" CEC cement, albeit less in scenario 2b where the clay is calcined in an electric oven powered by emissions-free electricity. However, as the lime-flux content is reduced, this advantage is overcome, and given that total CEC Clinker output will probably be restricted by total EAF capacity, it is likely that blending with calcined clay will remain advantageous. This prioritises further work on reducing the emissions of calcined clay production.

The costs of CEC clinker production are dominated by the costs of heating prior to RCP separation, and this depends on the price of electricity. When blending CEC clinker into a cement with calcined clay, the cost of the required calcined clay and anyhydrous calcium sulphate powder is approximately equal to those of the CEC Clinker but may reduce as the market for calcined clay expands.

Analysis of potential global scale of electric cement recycling

To estimate the global potential scale and abatement that could be delivered by the new process, we have built a simple model. It uses the International Energy Agency (IEA) scenario for future cement consumption as a baseline (IEA, 2009). Historical cement production is obtained from the US geological survey (USGS) with past clinker production from Andrew (2018). The clinker factor for 2020 is estimated to be 0.75, and is estimated to be close to one around 1980. Using the factors used in the previous section of analysis in this supplementary information, the impact of producing all cement as LC3 50 is first assessed. The future availability of calcium-rich RCP is then estimated to be the clinker production delayed by 50 years, assuming average building lives of 50 years (Dunant et al., 2021). Electricity emissions factors are assumed to decrease steadily to 30% of today's value by 2050. Future EAF recycling capacity is calculated using the IEA's iron and steel roadmap estimates (IEA, 2020b).

The resulting material production (of cement and clinker) and emissions arising from two scenarios are shown in Supplementary Table 9 and presented in figure 3C in the main paper: firstly, using only the available EAF capacity to process as much RCP as it can accept and secondly, assuming that additional dedicated EAF capacity is constructed to produce clinker but not steel and ensure that more of the available RCP is processed. In the attached spreadsheet, more ambitious scenarios are also computed for comparison.

Date	Cement production	Clay production	Clay electrification factor	Clinker factor	Clinker production	Clinker electrification factor	RCP Production	RCP factor	Clinker emissions	Energy Efficiency	Fuel CO2 factor	Combustion emissions	Electricity emissions	Electricity factor	Total emissions	Energy emissions	Steel production	EAF fraction of steel production	Total slag availability
**Baseline – Historical emissions**																			
1960	317			0.95	301				154	0.25	0.12	302	4	0.022	460	306			
1970	571			0.95	543				277	0.25	0.10	445	7	0.022	729	452			
1980	887			0.87	772				394	0.28	0.10	553	10	0.019	957	563			
1990	1,300			0.82	1,066				545	0.29	0.09	722	12	0.017	1,279	735	800		
2000	1,600			0.82	1,312				670	0.30	0.09	777	12	0.013	1,459	789			
2010	3,310			0.67	2,218				1,133	0.31	0.09	1,271	25	0.013	2,429	1,296	1,200		
2020	4,200			0.71	2,982				1,524	0.32	0.09	1,655	30	0.013	3,209	1,685	1,700	0.47	267
**Baseline – projected emissions**																			
2030	4,250			0.73	3,103				1,585	0.33	0.09	1,670	24	0.010	3,280	1,694			
2040	4,429			0.76	3,366				1,720	0.34	0.08	1,655	18	0.007	3,393	1,673			
2050	4,682			0.80	3,746				1,914	0.35	0.08	1,789	13	0.005	3,717	1,803			
2060	4,720			0.85	4,012				2,050	0.37	0.08	1,813	8	0.003	3,871	1,821			
**LC3-max – 90% LC3 2050**																			
2030	4,250	425	0	0.70	2,975		887	0.00	1,520	0.33	0.09	1,602	24	0.010	3,146	1,626	2,100	0.57	400
2040	4,429	886	0.5	0.60	2,657		1,300	0.00	1,358	0.34	0.08	1,307	22	0.007	2,687	1,329	2,250	0.76	567
2050	4,682	1,405	1	0.56	2,622		1,600	0.00	1,340	0.35	0.08	1,253	24	0.005	2,616	1,276	2,510	0.84	700
2060	4,720	1,416	1	0.50	2,360		3,310	0.00	1,206	0.37	0.08	1,066	14	0.003	2,287	1,081	2,600	0.87	750
**CEC-slow – 90% LC3, 35% CEC 2050**																			
2030	4,250	425	0	0.70	2,886		887	0.10	1,475	0.33	0.09	1,554	24	0.010	3,053	1,578	2,100	0.57	400
2040	4,429	886	0.5	0.60	2,332		1,300	0.25	1,192	0.34	0.08	1,147	20	0.007	2,359	1,167	2,250	0.76	567
2050	4,682	1,405	1	0.56	2,062		1,600	0.35	1,054	0.35	0.08	985	17	0.005	2,056	1,002	2,510	0.84	700
2060	4,720	1,416	1	0.50	1,610		3,310	0.23	823	0.37	0.08	728	11	0.003	1,561	738	2,600	0.87	750
**CEC-fast – 90% LC3, 44% (max) CEC 2050**																			
2030	4,250	425	0	0.70	2,842		887	0.15	1,452	0.33	0.09	1,391	24	0.010	2,868	1,415	2,100	0.57	400
2040	4,429	886	0.5	0.60	2,091		1,300	0.44	1,068	0.34	0.08	935	20	0.007	2,023	955	2,250	0.76	567
2050	4,682	1,405	1	0.55	1,875		1,600	0.44	958	0.35	0.08	814	18	0.005	1,790	832	2,510	0.84	700
2060	4,720	1,416	1	0.50	1,610		3,310	0.23	823	0.37	0.08	661	11	0.003	1,495	672	2,600	0.87	750
**Electric clinker-slow – 90% LC3, 65% CEC 2050**																			
2030	4,250	425	0	0.70	2,842	0.20	887	0.15	1,234	0.33	0.09	1,224	27	0.010	2,486	1,251	2,100	0.57	400
2040	4,429	886	0.5	0.60	2,007	0.35	1,300	0.50	513	0.34	0.08	642	26	0.007	1,180	667	2,250	0.76	567
2050	4,682	1,405	1	0.56	1,582	0.50	1,600	0.65	283	0.35	0.08	378	27	0.005	688	405	2,510	0.84	700
2060	4,720	1,416	1	0.50	-	0.65	3,310	0.75	-	0.37	0.08	-	16	0.003	16	16	2,600	0.87	750
**Electric clinker-fast - Electric clinker-slow – 90% LC3, 75% CEC 2050**																			
2030	4,250	425	0	0.70	2,798	0.30	887	0.20	1,144	0.33	0.06	707	29	0.010	1,879	736	2,100	0.57	400
2040	4,429	886	0.5	0.60	2,091	0.50	1,300	0.44	603	0.34	0.06	353	27	0.007	983	380	2,250	0.76	567
2050	4,682	1,405	1	0.56	1,422	0.70	1,600	0.75	182	0.35	0.05	127	28	0.005	337	155	2,510	0.84	700
2060	4,720	1,416	1	0.50	-	1.00	3,310	0.75	-	0.37	0.04	-	16	0.003	16	16	2,600	0.87	750

All quantities in Mt ; All emissions in Mt CO2 ; Fuel factors in kg CO2/MJ ; Electricity factors in kg CO2/MJ ; RCP factor, clay electrification factor, clinker factor, energy efficiency, EAF fraction unitless

Supplementary Table 9: Global emissions and scale scenarios to assess the impact of various interventions in cement production. (Data for 1960-2020 from USGS, forecast for 2030-2050 from IEA, 2060 values extrapolated by the authors. Clinker factors from Andrew, 2018)

Additional references

Andrew, R. M. (2018). Global CO_2 emissions from cement production, 1928–2017. *Earth System Science Data*, 10(4), 2213-2239.

Akan, M. Ö. A., Dhavale, D. G., & Sarkis, J. (2017). Greenhouse gas emissions in the construction industry: An analysis and evaluation of a concrete supply chain. *Journal of Cleaner Production*, *167*, 1195-1207.

BGS (2022), Brick-Clay, British Geological Survey Mineral Planning Factsheet, https://nora.nerc.ac.uk/id/eprint/532490/ accessed 10 Jan 2023

Bishnoi, S., Maity, S., Mallik, A., Joseph, S. & Krishnan, S. (2014) Pilot scale manufacture of limestone calcined clay cement: the Indian experience. *Indian Concr. J* **88 (6)**, 22–28

Czigler, T., Reiter, S., Schulze, P., & Somers, K. (2020). Laying the foundation for zero-carbon cement. *McKinsey & Company*, 9. (https://www.mckinsey.com/industries/chemicals/our-insights/laying-the-foundation-for-zero-carbon-cement accessed 13 Feb 2023)

Díaz, Y. C., Berriel, S. S., Heierli, U., Favier, A. R., Machado, I. R. S., Scrivener, K. L., ... & Habert, G. (2017). Limestone calcined clay cement as a low-carbon solution to meet expanding cement demand in emerging economies. *Development Engineering*, *2*, 82-91.

Dunant, C. F., Shah, T., Drewniok, M. P., Craglia, M., & Cullen, J. M. (2021). A new method to estimate the lifetime of long-life product categories. *Journal of Industrial Ecology*, **25(2)**, 321-332.

EPA (2009) Technical Support Document for the Lime Sector: Proposed Rule for Mandatory Reporting of Greenhouse Gases, downloaded from https://www.epa.gov/sites/default/files/2015-07/documents/subpartstsd.pdf, accessed 10 Jan 2023

FT (2021), Shipping: carbon conundrum cramps cycle, Opinion Lex, 11 May 2021, accessed 17 Feb 2023.

Global Cement (2011), Cement price trends in the UK, https://www.globalcement.com/magazine/articles/643-cement-price-trends-in-the-uk, accessed 21 Feb 2023.

Hanein, T., Galvez-Martos, J. L., & Bannerman, M. N. (2018). Carbon footprint of calcium sulfoaluminate clinker production. *Journal of Cleaner Production*, *172*, 2278-2287.

IEA (International Energy Agency) (2009), Cement Technology Roadmap 2009: Carbon emissions reductions up to 2050; 2009 https://www.iea.org/reports/cement-technology-roadmap-carbon-emissions-reductions-up-to-2050 accessed 10 Nov 2023.

IEA (2020). World Energy Outlook 2020. vol, 2050, 1-461.

IEA (International Energy Agency) (2020b), Iron and Steel Technology Roadmap: Carbon emissions reductions up to 2050; 2020, https://www.iea.org/reports/iron-and-steel-technology-roadmap# accessed 10 Nov 2023.

IPCC (2014). Contribution of working group III to the fifth assessment report of the Intergovernmental Panel on Climate Change. Mitigation of climate change.

Jansen, D., Goetz-Neunhoeffer, F., Stabler, C., Neubauer, J. (2011). A remastered external standard method applied to the quantification of early OPC hydration. *Cement and Concrete Research,* 41, 602-608.

Meier, A., Bonaldi, E., Cella, G. M., & Lipinski, W. (2005). Multitube rotary kiln for the industrial solar production of lime, *Journal of Solar Energy Engineering, Transactions of the ASME*, **127**, 386-395

Prajapati, R., Gettu, R., & Singh, S. (2021). Thermomechanical beneficiation of recycled concrete aggregates (RCA). *Construction and Building Materials*, 310, 125200.

Proske, T., Hainer, S., Rezvani, M., & Graubner, C. (2016). Eco-friendly concretes with reduced water and cement content: mix design principles and experimental tests. *Constr. Build. Mater. J*, *67*, 63-87.

Rivero, A. J., Sathre, R., & Navarro, J. G. (2016). Life cycle energy and material flow implications of gypsum plasterboard recycling in the European Union. *Resources, Conservation and Recycling*, *108*, 171-181.

Scrivener, K., Martirena, F., Bishnoi, S., & Maity, S. (2018). Calcined clay limestone cements (LC3). *Cement and Concrete Research*, 114, 49-56.

Snellings, R., Bazzoni, A., Scrivener, K., (2014). The existence of amorphous phase in Portland cements: Physical factors affecting Rietveld quantitative phase analysis. *Cement and Concrete Research*, 59, 139-146.

USGS, Geological Survey, Interior Department, & Mines Bureau. (various years). Minerals Yearbook: Metals and Minerals. US Government Printing Office
